# Supplementary material for: Clinical utility of tumour mutational burden on efficacy of immune checkpoint inhibitors in malignant solid tumours: protocol for a systematic review and meta-analysis
Source: BMJ Open. 2022 Aug 4;12(8):e058692. doi: 10.1136/bmjopen-2021-058692 (PMC9358952; doi:10.1136/bmjopen-2021-058692)
Supplement: Supplementary data [file bmjopen-2021-058692supp001.pdf]

Search strategies and results of 5 databases

Database: PubMed, OVID, Embase, the Cochrane Central Register of Controlled Trials Library databases, Web of Science.

Data Run: 01/07/2022

database	Search strategies	results
Pubmed	#1 "Immune Checkpoint Inhibitors" [Mesh Terms]	6109
	#2 "immunotherap*"[Title/Abstract] OR "immune checkpoint inhibit*"[Title/Abstract] OR "ICI"[Title/Abstract] OR "ICIs"[Title/Abstract] OR "immune checkpoint block*"[Title/Abstract] OR "ICB"[Title/Abstract] OR "ICBs"[Title/Abstract] OR "pembrolizumab"[Title/Abstract] OR "avelumab"[Title/Abstract] OR "nivolumab"[Title/Abstract] OR "durvalumab"[Title/Abstract] OR "tremelimumab"[Title/Abstract] OR "atezolizumab"[Title/Abstract] OR "Ipilimumab"[Title/Abstract] OR "Cemiplimab"[Title/Abstract] OR "tiragolumab"[Title/Abstract] OR "Dostarlimab*"[Title/Abstract] OR "Camrelizumab"[Title/Abstract] OR "PD-1"[Title/Abstract] OR "programmed death 1"[Title/Abstract] OR "PD-L1"[Title/Abstract] OR "programmed death-ligand 1"[Title/Abstract] OR "PD-1/PD-L1"[Title/Abstract] OR "anti–PD-1/anti–PD-L1"[Title/Abstract] OR "CTLA-4"[Title/Abstract] OR "Cytotoxic T-lymphocyte antigen 4"[Title/Abstract]	164405
	#3 #1 OR #2	164676
	#4 "Carcinoma"[Title/Abstract] OR "Neoplasms"[Title/Abstract] OR "Cancer"[Title/Abstract] OR "Tumour" [Title/Abstract] OR "Tumor"[Title/Abstract]	3285875
	#5 #3 and #4	102540
	#6 "mutation burden"[Title/Abstract] OR "mutational burden"[Title/Abstract] OR "mutation load"[Title/Abstract] OR "mutational load"[Title/Abstract] OR "TMB"[Title/Abstract] OR "TML"[Title/Abstract]	10061
	#7 #5 and #6	3268
OVID	#1 exp Immune Checkpoint Inhibitors/	14572
	#2 ((immunotherap*) or (immune checkpoint inhibit*) or (ICI) or (immune checkpoint inhibit*) or (ICIs) or (immune checkpoint block*) or (ICB) or (ICBs) or (pembrolizumab) or (avelumab) or (nivolumab) or (durvalumab) or (tremelimumab) or (atezolizumab) or (Ipilimumab) or (Cemiplimab) or (tiragolumab) or (Dostarlimab) or (Camrelizumab) or (PD-1) or (programmed death 1) or (PD-L1) or (programmed death-ligand 1) or (anti-PD-1) or (anti-PD-L1) or (CTLA-4) or (Cytotoxic T-lymphocyte antigen 4)).tw.	153834
	#3 #1 OR #2	156787
	#4 ((Carcinoma) or (Neoplasms) or (Cancer) or (Tumour) or (Tumor)).tw.	3136257
	#5 #3 and #4	94825
	#6 ((mutation burden) or (mutational burden) or (mutation load) or (mutational load) or (TMB) or (TML)).tw.	9467
	#7 #5 and #6	2963
Embase	#1 'immunotherap*:ab,ti OR 'immune checkpoint inhibit*':ab,ti OR 'ici':ab,ti OR 'icis':ab,ti OR 'immune checkpoint block*':ab,ti OR 'icb':ab,ti OR 'icbs':ab,ti OR 'pembrolizumab':ab,ti OR 'avelumab':ab,ti OR 'nivolumab':ab,ti OR 'durvalumab':ab,ti OR 'tremelimumab':ab,ti OR 'atezolizumab':ab,ti OR 'ipilimumab':ab,ti OR 'cemiplimab':ab,ti OR 'tiragolumab':ab,ti OR 'dostarlimab*':ab,ti OR 'camrelizumab':ab,ti OR 'pd-1':ab,ti OR 'programmed death 1':ab,ti OR 'pd-l1':ab,ti OR 'programmed death-ligand 1':ab,ti OR 'pd-1/pd-l1':ab,ti OR 'anti–pd-1/anti–pd-l1':ab,ti OR 'ctla-4':ab,ti OR 'cytotoxic t-lymphocyte atigen 4':ab,ti	251032
	#2 'Carcinoma':ab,ti OR 'Neoplasms':ab,ti OR 'Cancer':ab,ti OR 'Tumour' ab,ti OR 'Tumor':ab,ti	1809315
	#3 #1 and #2	101253
	#4 'mutation burden':ab,ti OR 'mutational burden':ab,ti OR 'mutation load':ab,ti OR 'mutational load':ab,ti OR 'TMB':ab,ti OR 'TML':ab,ti	16290
	#5 #3 and #4	5654
Cochrane	#1 ("immunotherap* OR immune checkpoint inhibit* OR ICI OR ICIs OR immune checkpoint block* OR ICB OR ICBs OR pembrolizumab OR avelumab OR nivolumab OR durvalumab OR tremelimumab OR atezolizumab OR Ipilimumab OR Cemiplimab OR tiragolumab OR Dostarlimab* OR Camrelizumab	21080

	OR PD-1 OR programmed death 1 OR PD-L1 OR programmed death-ligand 1 OR PD-1/PD-L1 OR anti–PD-1/anti–PD-L1 ORCTLA-4 OR Cytotoxic T-lymphocyte antigen 4):ti,ab,kw<br>#2 （Carcinoma OR Neoplasms OR Cancer OR Tumour OR Tumor):ti,ab,kw<br>#3 #1 and #2<br>#4 (mutation burden OR mutational burden OR mutation load OR mutational load OR TMB OR TML):ti,ab,kw<br>#5 #3 and #4	242648<br>13396<br>1495<br>460
web of science	#1 (TI=(“immunotherap*” OR “immune checkpoint inhibit*” OR “ICI” OR "ICIs" OR “immune checkpoint block*” OR "ICB" OR "ICBs" OR “pembrolizumab” OR “avelumab” OR “nivolumab” OR “durvalumab” OR “tremelimumab” OR “atezolizumab” OR “Ipilimumab” OR “Cemiplimab” OR “tiragolumab” OR “Dostarlimab*” OR “Camrelizumab” OR “PD-1” OR “programmed death 1” OR “PD-L1” OR “programmed death-ligand 1” OR “PD-1/PD-L1” OR “anti–PD-1/anti–PD-L1” OR “CTLA-4” OR “Cytotoxic T-lymphocyte atigen 4”)) OR AB=(“immunotherap*” OR “immune checkpoint inhibit*” OR “ICI” OR "ICIs" OR “immune checkpoint block*” OR "ICB" OR "ICBs" OR “pembrolizumab” OR “avelumab” OR “nivolumab” OR “durvalumab” OR “tremelimumab” OR “atezolizumab” OR “Ipilimumab” OR “Cemiplimab” OR “tiragolumab” OR “Dostarlimab*” OR “Camrelizumab” OR “PD-1” OR “programmed death 1” OR “PD-L1” OR “programmed death-ligand 1” OR “PD-1/PD-L1” OR “anti–PD-1/anti–PD-L1” OR “CTLA-4” OR “Cytotoxic T-lymphocyte antigen 4”)<br>#2 (TS = ("Carcinoma" OR " Neoplasms" OR " Cancer" OR " Tumour" OR " Tumor")) OR AB = ("Carcinoma" OR " Neoplasms" OR " Cancer" OR " Tumour" OR " Tumor")<br>#3 #1 and #2<br>#4 (TS = ("mutation burden" OR "mutational burden" OR "mutation load" OR "mutational load" OR "TMB" OR "TML")) OR AB = ("mutation burden" OR "mutational burden" OR "mutation load" OR "mutational load" OR "TMB" OR "TML")<br>#5 #3 and #4	191683<br>4045332<br>111505<br>13058<br>3510
