# Supplementary material for: Clinical utility of tumour mutational burden on efficacy of immune checkpoint inhibitors in malignant solid tumours: protocol for a systematic review and meta-analysis
Source: BMJ Open. 2022 Aug 4;12(8):e058692. doi: 10.1136/bmjopen-2021-058692 (PMC9358952; doi:10.1136/bmjopen-2021-058692)
Supplement: Author's manuscript [file bmjopen-2021-058692.draft_revisions.pdf]

BMJ Open

BMJ Open is committed to open peer review. As part of this commitment we make the peer review history of every article we publish publicly available.

When an article is published we post the peer reviewers' comments and the authors' responses online. We also post the versions of the paper that were used during peer review. These are the versions that the peer review comments apply to.

The versions of the paper that follow are the versions that were submitted during the peer review process. They are not the versions of record or the final published versions. They should not be cited or distributed as the published version of this manuscript.

BMJ Open is an open access journal and the full, final, typeset and author-corrected version of record of the manuscript is available on our site with no access controls, subscription charges or pay-per-view fees (http://bmjopen.bmj.com).

If you have any questions on BMJ Open's open peer review process please email info.bmjopen@bmj.com

BMJ Open

The Clinical Utility of Tumor Mutational Burden on Efficacy of Immune Checkpoint Inhibitors in Solid tumor: Protocol for A Systematic Review and Meta-analysis

Journal:	*BMJ Open*
Manuscript ID	bmjopen-2021-058692
Article Type:	Protocol
Date Submitted by the Author:	26-Oct-2021

Complete List of Authors:	Xiang, Xuemei; People's Liberation Army The General Hospital of Western Theater Command, Basic Medical Laboratory<br>Li, Yunming; People's Liberation Army The General Hospital of Western Theater Command, Department of Information; Southwest Jiaotong University, Department of Statistics<br>Guo, Wang; Southwest Jiaotong University, Department of Statistics; People's Liberation Army The General Hospital of Western Theater Command, Department of Information<br>Zhou, Pengfei; Southwest Medical University; People's Liberation Army The General Hospital of Western Theater Command, Department of Information

Keywords:	Adult oncology < ONCOLOGY, Oncogenes < ONCOLOGY, IMMUNOLOGY

SCHOLARONE™ Manuscripts

The Clinical Utility of Tumor Mutational Burden on Efficacy of Immune Checkpoint Inhibitors in Solid tumor: Protocol for A Systematic Review and Meta-analysis

Xuemei Xiang[1]MD, Yunming Li*[2,3] PhD, Wang Guo[3,2]Mr, Pengfei Zhou[4,2]Mr

1. Basic Medical Laboratory, The General Hospital of Western Theater Command， Chengdu, Sichuan Province, 610083, China
2. Department of Information, The General Hospital of Western Theater Command, Chengdu, Sichuan Province, 610083, China.
3.Department of Statistics, College of Mathematics, Southwest Jiaotong University, Chengdu, Sichuan Province, 610031, China.
4.School of Public Health, Southwest Medical University, Luzhou, Sichuan Province, 646000, China.

***Corresponding author:** Yunming Li, Male, Ph.D. in epidemiology and health statistics, Postdoctoral in clinical medicine, deputy director technician. Email: lee3082@sina.com, Telephone number: 18908007958. Postal address: 270 Tianhuan Road, Jinniu District, Chengdu, Sichuan, China.

Word count

2045

Keywords: Tumor Mutational Burden; Immune Checkpoint Inhibitors; Solid tumor; Systematic Review

For peer review only

ABSTRACT

Introduction: A major advance in solid malignancies treatment is the development of immune checkpoint inhibitors (ICIs) that have produced durable responses and improved survival. However, the therapeutic effect of ICIs has great heterogeneity in cancer patients. We conduct a systematic review and meta-analysis to evaluate the predictive value of tumor mutation burden (TMB) on efficacy of ICIs.

Methods and analysis: Systematic literature search was conducted on PubMed, OVID, Web of Science, Embase and the Cochrane Central Register of Controlled Trials Library up to 31 October 2021. The comparison on efficiency of ICIs between TMB high group and TMB low group, which was measured in terms of odds ratio (OR) of objective response rate/overall response rate (ORR), and hazard ratio (HR) of progression-free survival (PFS) and overall survival (OS). OR of ORR, HR of PFS and OS were estimated by inverse variance weighted fixed-effects model ($I^2 \leq 50\%$) or DerSimonian-Laird random-effects model ($I^2 > 50\%$). In addition, heterogeneity analysis, sensitivity analysis, publication bias and subgroup analysis were conducted. We plan to conduct a subgroup analysis on age, gender, area, number of patients (High/Low TMB), tumor size, stage, TMB sequencing method, type of immunotherapy or follow-up period. Moreover, fractional polynomial regression was conducted to investigate the dose-response relationship between TMB cutoffs and efficacy of ICIs.

Ethics and dissemination: Ethical approval and informed consent are not required, as the study will be a literature review and will not involve direct contact with patients or alterations to patient care. This systematic review is anticipated to be finished in December 2022, and the results will be published in a peer-reviewed journal.

PROSPERO registration number: CRD42021262480.

For peer review only

Article Summary

Strengths and limitations of this study

► This is an update comprehensive systematic review focused on tumor mutation burden and efficacy of ICIs for the prognosis of patients with solid tumor.

► We plan to conduct a comprehensive subgroup analysis of association between TMB and efficiency of ICIs, including age, gender, area, number of patients (High/Low TMB), tumor size, stage, TMB sequencing method, type of immunotherapy or follow-up period.

► We will focused on the long-term efficacy of ICIs in patients with solid tumor.

► We searched databases of English, while other languages may be ignored.

For peer review only - http://bmjopen.bmj.com/site/about/guidelines.xhtml

Introduction

ICIs have been identified to improve response and survival in diverse solid tumors and hematologic malignancies. However, the efficacy seems satisfactory in some patients, while others do not,[1-6] suggesting eligible biomarkers are required to identify subgroups appropriate for cancer immunotherapy. At present, scientists have recognized several candidate biomarkers, such as programmed cell death ligand 1 (PD-L1), transcriptomic and epigenetic signatures, tumorinfiltrating lymphocytes (TILs), oncogenic driver mutations and mismatch repair deficiency (dMMR).[7] Among them, TMB is likely to be a promising biomarker. TMB is broadly defined as the number of somatic mutations per megabase of interrogated genomic sequence. TMB is a continuous variable and variability of TMB (ranging from 0.001/Mb to more than 1000/Mb) has been observed across and within cancer types.[8 9] It was suggested that a higher TMB increase the likelihood of generating immunogenic tumor neoantigens recognized by the host immune system.[10-12]

Retrospective evidence suggests that TMB can predict the efficacy of ICIs, and recent U.S. Food and Drug Administration (FDA) approval of pembrolizumab for the TMB-high tumor subgroup. But, the predict value seems inconsistent in patients with different tumor types, this may be associated with the degree of variability in TMB. Current investigations indicate that some cancer types have less variability in TMB such as lung and head and neck cancers, and some having greater variability such as colon, bladder, and uterine cancers.[13] Studies are attempting to validate the long-term oncologic impact of TMB. Despite a number of studies uncovering powerful forecasting capability of TMB on efficacy of ICIs, however, negative results are also reported, especially in long-term survival.[14-16] Although there are three meta-analyses reporting the predictive value of TMB.[17-19] The sample size of the first two studies is small and the subgroup analysis is incomplete.[17,18] 29 studies were included in the latest one in 2019, with a total of 4431 patients,[19] however, there is also a lack of support of long-term efficacy of all types of tumors due to the insufficient number of studies and patients, and it is not enough to seek out the best threshold of TMB, and studies are far from enough to make a convincing conclusion in small cell lung cancer (SCLC). Moreover, most of the studies done in PD-(L)1 monotherapy, and the research on combined therapy is also insufficient.

Hence, we did an update comprehensive systematic review and meta-analysis to evaluate the influence of tumor mutation burden on efficacy of ICIs in solid tumors, and conduct overall subgroup analyses to identify potential source of heterogeneity.

Method

MATERIAL AND METHODS

We submitted this study protocol to PROSPERO (CRD42021262480). Methods for this systematic review and pairwise meta- analysis follow the Preferred Reporting Items for Systematic Review and Meta- Analysis Protocols.[20 21] As this is a systematic literature research, ethical approval is waived.

Inclusion and exclusion criteria

For peer review only - http://bmjopen.bmj.com/site/about/guidelines.xhtml

We will include all prospective or retrospective studies that meet the following criteria:

1. Cohort studies or clinical trials assessed inhibitors of PD-1/PD-L1, Cytotoxic T Lymphocyte-associated Antigen-4 (CTLA-4), or their combination, or with chemotherapy, in patients with solid tumors, and the efficiency of therapy was evaluated by TMB which had cut-off. value; either alone or combined with each other or with chemotherapy.
2. OR of ORR, or HR of PFS or OS, and their 95% confidence intervals (95% CI) were given in the article, or sufficient data was available to calculate them.
3. Comparison: ICIs treatment, within High TMB Group or Low TMB Group.
4. Outcomes:

► Association between TMB and response rate of ICIs in all kinds of solid tumor types, including OS, PFS, DFS, RFS, DSS, et.al.

► Association of subgroup analysis between TMB and efficiency of ICIs,, including age, gender, area, number of patients (High/Low TMB), tumor size, stage, TMB sequencing method, type of immunotherapy or follow-up period.

► Correlations between TMB and clinicopathological features, such as tumor size, stage, and metastasis.

Exclusion criteria:

1. Review, comments, case reports, nonhuman study.
2. The study did not contain a control groups and analysis.
3. The data needed to be extracted in the study is incomplete.

Search strategy

From inception to 31 October 2021, PubMed, Ovid, Web of Science, Embase and the Cochrane Central Register of Controlled Trials will be searched using the MeSH terms “Tumor Burden”, “ Tumor Load”, “ Tumor Weight” and “ Immune Checkpoint Inhibitor”, “ Immune Checkpoint Inhibition”, “ Immune Checkpoint Blockers”, “ Immune Checkpoint Blockade”, “PD-1”, “PD-L1”, “CTLA-4” or the name of the drugs (ie, nivolumab, pembrolizumab, atezolizumab, durvalumab, avelumab, ipilimumab, tremelimumab) and the related keywords “ Tumor Mutational Burden” or “ Tumor Mutation Burden”. The languages will not be limited in our search strategy. The search strategy of Ovid is presented in table 1.

Data abstraction

XMX and WG will independently assess the eligibility of reports from the title and/or abstract. A third reviewer, YML, will join them to resolve any disagreements. Studies that meet the inclusion criteria will be selected for further analysis. For included studies, we will ask for the original data from corresponding authors for diagnosis and prognosis analysis. The following information will be extracted from each study: first author, study design, year of publication, median age, gender, TMB sequencing method, follow-up period, type of cancer, tumor size, stage, type of immunotherapy, TMB

For peer review only - http://bmjopen.bmj.com/site/about/guidelines.xhtml

cutoff， number of Patients (High/Low TMB) , area of patients, outcomes (ORR, PFS, OS, et.al) and their value. When duplicate publications were identified, the most comprehensive one was included.

Assessment of risk of bias in included studies and study quality

Two review authors (YML and WG) will independently assess the risk of bias for each study using the Newcastle-Ottawa Scale(NOS). NOS was adopted to assess the quality of studies included.[22] The total score ranged from 0 to 9, as 8–9 points indicated high quality of a study, five to seven points indicated medium quality, and studies with points lower than five showed poor quality.

Assessment of publication bias

We plan to use the funnel plot and Egger's test to evaluate the potential publication bias by R-4.0.2 , only if at least 10 studies are included. P<0.05 will be considered to indicate significant publication bias.

Assessment of heterogeneity

The χ^2 test will be used to examine heterogeneity in pooling analysis. Heterogeneity is considered to be statistically significant when P <0.10 in these qualitative tests. We plan to use the I^2 test to estimate the proportion of total variation across studies that is attributable to heterogeneity rather than chance, with values of 25%, 50% and 75% indicating low, moderate and high inconsistency, respectively. To determine the source of heterogeneity, we will conduct a meta- regression on different factors within R-4.0.2. We also plan to conduct a subgroup analysis on age, gender, area, number of patients (High/Low TMB), tumor size, stage, TMB sequencing method, type of immunotherapy or follow-up period.

Sensitivity analysis

To determine the robustness of the pooled results, we will conduct a sensitivity analysis by examining individual studies on estimated effects using R-4.0.2.

Data synthesis

The primary endpoint of the meta-analysis was the comparison on efficiency of ICIs between TMB high group and TMB low group, which was measured in terms of OR of ORR, and HR of PFS and OS. Heterogeneity among individual studies was evaluated by the Q test; I^2> 50% and/or P<0.10 indicated significant heterogeneity.[23] Pooled OR or HR with Z test was calculated by DerSimonian-Laird random-effects model when significant heterogeneity was identified, otherwise inverse variance weighted fixed-effects model was adopted. In addition, funnel plots were constructed, and Begg's test and Egger's test were performed to evaluate publication bias (P<0.10 was considered to be visible publication bias). Besides, sensitivity analysis was used to test the stability of the results in the meta-analysis. To further explore variation of effect of TMB on immunotherapy efficiency, subgroup analyses stratified by cancer type, area of patients, TMB sequencing method, class of ICIs, and line of therapy were conducted. Moreover, to investigate the dose-response relationship between

For peer review only - http://bmjopen.bmj.com/site/about/guidelines.xhtml

For peer review only

TMB cutoffs and efficacy of ICIs, fractional polynomial regression (two degree) was conducted on studies of no <50 patients. To note, total mutation burden detected by whole exome sequencing (WES) was converted to mutations per megabase using a linear transformation.[24] Furthermore, we evaluated ORR by TMB and PD-L1 expression after layering each other in studies which the two could be both acquired. R-4.0.2 was used for analyses mentioned above.

Discussion

The rationale for the association between TMB and benefit from immunotherapy is based on the hypothesis that tumor mutation-specific neoantigens can be displayed on major histocompatibility complexes (MHC) on the tumor cell surface, and then recognized by tumor infiltrating T-cells, accordingly, a higher TMB will generate more neoantigens that can then trigger intratumoral T-cells whose ability to attack and destroy tumor cells is enabled by ICIs.[10 12]

As a new biomarker, there is an urgent need to harmonize and standardize TMB measurement, testing platforms and reporting of TMB. Various strategies to optimize TMB as a predictive biomarker of ICIs are being explored. Larger TMB data sets and clinical outcomes of patients treated with ICIs will help to optimize TMB cut-off values for specific cancer types, and it is possible to extend the approval of immunotherapy to a larger patient population. In addition, the combination of TMB with other potential biomarkers and computational assistance paves the way for accurate immunotherapy.

PATIENT AND PUBLIC INVOLVEMENT

As the study is a protocol of meta- analysis based on previously published literature, the primary patient data will not be collected. Patient or public will not be involved in the study design, recruitment and data analysis.

Contributors: XMX, WG and YML conceived the study and drafted the manuscript. XMX and YML registered the protocol review in the PROSPERO database. YML and WG designed the search strategy. PFZ, WG and YML formed the data synthesis and analysis plan. XMX and YML supervised this study and revised the manuscript.

Founding: Military Medical Research Project, the General Hospital of Western Theater Command, Chinese People's Liberation Army (2019ZY10, 2019ZY04); 2021 Basic Research Cultivation Project of the Central Universities (2682021ZTPY018)

Competing interests: None declared.

Patient consent for publication: Not required.

Provenance and peer review: Not commissioned; externally peer reviewed.

References

1. Schadendorf D, Hodi FS, Robert C, et al. Pooled Analysis of Long-Term Survival Data From Phase II and Phase III Trials of Ipilimumab in Unresectable or Metastatic Melanoma. *Journal of clinical oncology : official journal of the American*

For peer review only - http://bmjopen.bmj.com/site/about/guidelines.xhtml

Society of Clinical Oncology 2015;33(17):1889-94. doi: 10.1200/jco.2014.56.2736 [published Online First: 2015/02/11]

2. Overman MJ, Lonardi S, Wong KYM, et al. Durable Clinical Benefit With Nivolumab Plus Ipilimumab in DNA Mismatch Repair-Deficient/Microsatellite Instability-High Metastatic Colorectal Cancer. *Journal of clinical oncology : official journal of the American Society of Clinical Oncology* 2018;36(8):773-79. doi: 10.1200/jco.2017.76.9901 [published Online First: 2018/01/23]
3. Motzer RJ, Tannir NM, McDermott DF, et al. Nivolumab plus Ipilimumab versus Sunitinib in Advanced Renal-Cell Carcinoma. *The New England journal of medicine* 2018;378(14):1277-90. doi: 10.1056/NEJMoa1712126 [published Online First: 2018/03/22]
4. Rosenberg JE, Hoffman-Censits J, Powles T, et al. Atezolizumab in patients with locally advanced and metastatic urothelial carcinoma who have progressed following treatment with platinum-based chemotherapy: a single-arm, multicentre, phase 2 trial. *Lancet (London, England)* 2016;387(10031):1909-20. doi: 10.1016/s0140-6736(16)00561-4 [published Online First: 2016/03/10]
5. Wolchok JD, Kluger H, Callahan MK, et al. Nivolumab plus ipilimumab in advanced melanoma. *The New England journal of medicine* 2013;369(2):122-33. doi: 10.1056/NEJMoa1302369 [published Online First: 2013/06/04]
6. Hodi FS, O'Day SJ, McDermott DF, et al. Improved survival with ipilimumab in patients with metastatic melanoma. *The New England journal of medicine* 2010;363(8):711-23. doi: 10.1056/NEJMoa1003466 [published Online First: 2010/06/08]
7. Havel JJ, Chowell D, Chan TA. The evolving landscape of biomarkers for checkpoint inhibitor immunotherapy. *Nature reviews Cancer* 2019;19(3):133-50. doi: 10.1038/s41568-019-0116-x [published Online First: 2019/02/14]
8. Alexandrov LB, Nik-Zainal S, Wedge DC, et al. Signatures of mutational processes in human cancer. *Nature* 2013;500(7463):415-21. doi: 10.1038/nature12477 [published Online First: 2013/08/16]
9. Chalmers ZR, Connelly CF, Fabrizio D, et al. Analysis of 100,000 human cancer genomes reveals the landscape of tumor mutational burden. *Genome medicine* 2017;9(1):34. doi: 10.1186/s13073-017-0424-2 [published Online First: 2017/04/20]
10. Chan TA, Wolchok JD, Snyder A. Genetic Basis for Clinical Response to CTLA-4 Blockade in Melanoma. *The New England journal of medicine* 2015;373(20):1984. doi: 10.1056/NEJMc1508163 [published Online First: 2015/11/13]
11. Van Allen EM, Miao D, Schilling B, et al. Genomic correlates of response to CTLA-4 blockade in metastatic melanoma. *Science (New York, NY)* 2015;350(6257):207-11. doi: 10.1126/science.aad0095 [published Online First: 2015/09/12]
12. Rizvi NA, Hellmann MD, Snyder A, et al. Cancer immunology. Mutational landscape determines sensitivity to PD-1 blockade in non-small cell lung cancer. *Science (New York, NY)* 2015;348(6230):124-8. doi: 10.1126/science.aaa1348 [published Online First: 2015/03/15]
13. Merino DM, McShane LM, Fabrizio D, et al. Establishing guidelines to harmonize tumor mutational burden (TMB): in silico assessment of variation in TMB

For peer review only - http://bmjopen.bmj.com/site/about/guidelines.xhtml

quantification across diagnostic platforms: phase I of the Friends of Cancer Research TMB Harmonization Project. *Journal for immunotherapy of cancer* 2020;8(1) doi: 10.1136/jitc-2019-000147 [published Online First: 2020/03/29]
14. Tang B, Yan X, Sheng X, et al. Safety and clinical activity with an anti-PD-1 antibody JS001 in advanced melanoma or urologic cancer patients. *Journal of hematology & oncology* 2019;12(1):7. doi: 10.1186/s13045-018-0693-2 [published Online First: 2019/01/16]
15. Morrison C, Pabla S, Conroy JM, et al. Predicting response to checkpoint inhibitors in melanoma beyond PD-L1 and mutational burden. *Journal for immunotherapy of cancer* 2018;6(1):32. doi: 10.1186/s40425-018-0344-8 [published Online First: 2018/05/11]
16. Mishima S, Kawazoe A, Nakamura Y, et al. Clinicopathological and molecular features of responders to nivolumab for patients with advanced gastric cancer. *Journal for immunotherapy of cancer* 2019;7(1):24. doi: 10.1186/s40425-019-0514-3 [published Online First: 2019/02/02]
17. Kim JY, Kronbichler A, Eisenhut M, et al. Tumor Mutational Burden and Efficacy of Immune Checkpoint Inhibitors: A Systematic Review and Meta-Analysis. *Cancers* 2019;11(11) doi: 10.3390/cancers11111798 [published Online First: 2019/11/17]
18. Wu Y, Xu J, Du C, et al. The Predictive Value of Tumor Mutation Burden on Efficacy of Immune Checkpoint Inhibitors in Cancers: A Systematic Review and Meta-Analysis. *Frontiers in oncology* 2019;9:1161. doi: 10.3389/fonc.2019.01161 [published Online First: 2019/11/22]
19. Zhu J, Zhang T, Li J, et al. Association Between Tumor Mutation Burden (TMB) and Outcomes of Cancer Patients Treated With PD-1/PD-L1 Inhibitions: A Meta-Analysis. *Frontiers in pharmacology* 2019;10:673. doi: 10.3389/fphar.2019.00673 [published Online First: 2019/07/02]
20. Moher D, Shamseer L, Clarke M, et al. Preferred reporting items for systematic review and meta-analysis protocols (PRISMA-P) 2015 statement. *Systematic reviews* 2015;4(1):1. doi: 10.1186/2046-4053-4-1 [published Online First: 2015/01/03]
21. Shamseer L, Moher D, Clarke M, et al. Preferred reporting items for systematic review and meta-analysis protocols (PRISMA-P) 2015: elaboration and explanation. *BMJ (Clinical research ed)* 2015;350:g7647. doi: 10.1136/bmj.g7647 [published Online First: 2015/01/04]
22. Stang A. Critical evaluation of the Newcastle-Ottawa scale for the assessment of the quality of nonrandomized studies in meta-analyses. *European journal of epidemiology* 2010;25(9):603-5. doi: 10.1007/s10654-010-9491-z [published Online First: 2010/07/24]
23. Higgins JP, Thompson SG, Deeks JJ, et al. Measuring inconsistency in meta-analyses. *BMJ (Clinical research ed)* 2003;327(7414):557-60. doi: 10.1136/bmj.327.7414.557 [published Online First: 2003/09/06]
24. Chang H, Sasson A, Srinivasan S, et al. Bioinformatic Methods and Bridging of Assay Results for Reliable Tumor Mutational Burden Assessment in Non-Small-Cell Lung Cancer. *Molecular diagnosis & therapy* 2019;23(4):507-20. doi: 10.1007/s40291-019-00408-y [published Online First: 2019/06/30]

For peer review only - http://bmjopen.bmj.com/site/about/guidelines.xhtml

For peer review only

For peer review only - http://bmjopen.bmj.com/site/about/guidelines.xhtml

Table 1. Search strategy (OVID)

Item	Search strategy
1	Immune Checkpoint Inhibitor/ or Immune Checkpoint Inhibition/or Immune Checkpoint Blockers / or Immune Checkpoint Blockade / or PD-1 / or PD-L1/ or CTLA-4/ or nivolumab/ or pembrolizumab / or atezolizumab / or avelumab / or durvalumab/ or tremelimumab / or ipilimumab /
2	Tumor Burden/ or Tumor Load / or Tumor Weight
3	1 and 2
4	(solid tumor$).ti,ab,kw,tw.
5	3 and 4

For peer review only

For peer review only - http://bmjopen.bmj.com/site/about/guidelines.xhtml

Abbreviations:

ICIs= Immune Checkpoint Inhibitors

TMB = Tumor Mutation Burden

OR = Odds Ratio

ORR = Objective Response Rate /Overall Response Rate (ORR)

HR = Hazard Ratio

OS = Overall Survival

PFS = Progression-free Survival

DFS = Disease-free Survival

RFS = Recurrence-free Survival

DSS = Disease-specific Survival

SCLC = Small Cell Lung Cancer

FDA = U.S. Food and Drug Administration

CTLA-4 = Cytotoxic T Lymphocyte-associated Antigen-4

dMMR = Oncogenic Driver Mutations and Mismatch Repair Deficiency

PD-L1 = Programmed Cell Death Ligand 1

PD-1 = Programmed Cell Death 1

TILs = Tumorinfiltrating Lymphocytes

CI = Confidence Interval

NOS = Newcastle-Ottawa Scale

WES = Whole Exome Sequencing

MHC = Major Histocompatibility Complexes

RCT = Randomized Controlled Trial.

For peer review only

For peer review only - http://bmjopen.bmj.com/site/about/guidelines.xhtml

PRISMA 2020 Checklist

Section and Topic	Item #	Checklist item	Location where item is reported
**TITLE**			
Title	1	Identify the report as a systematic review.	1
**ABSTRACT**			
Abstract	2	See the PRISMA 2020 for Abstracts checklist.	2
**INTRODUCTION**			
Rationale	3	Describe the rationale for the review in the context of existing knowledge.	3
Objectives	4	Provide an explicit statement of the objective(s) or question(s) the review addresses.	3
**METHODS**			
Eligibility criteria	5	Specify the inclusion and exclusion criteria for the review and how studies were grouped for the syntheses.	3,4
Information sources	6	Specify all databases, registers, websites, organisations, reference lists and other sources searched or consulted to identify studies. Specify the date when each source was last searched or consulted.	4
Search strategy	7	Present the full search strategies for all databases, registers and websites, including any filters and limits used.	4
Selection process	8	Specify the methods used to decide whether a study met the inclusion criteria of the review, including how many reviewers screened each record and each report retrieved, whether they worked independently, and if applicable, details of automation tools used in the process.	4
Data collection process	9	Specify the methods used to collect data from reports, including how many reviewers collected data from each report, whether they worked independently, any processes for obtaining or confirming data from study investigators, and if applicable, details of automation tools used in the process.	4
Data items	10a	List and define all outcomes for which data were sought. Specify whether all results that were compatible with each outcome domain in each study were sought (e.g. for all measures, time points, analyses), and if not, the methods used to decide which results to collect.	3,4
	10b	List and define all other variables for which data were sought (e.g. participant and intervention characteristics, funding sources). Describe any assumptions made about any missing or unclear information.	3,4
Study risk of bias assessment	11	Specify the methods used to assess risk of bias in the included studies, including details of the tool(s) used, how many reviewers assessed each study and whether they worked independently, and if applicable, details of automation tools used in the process.	4
Effect measures	12	Specify for each outcome the effect measure(s) (e.g. risk ratio, mean difference) used in the synthesis or presentation of results.	3,4
Synthesis methods	13a	Describe the processes used to decide which studies were eligible for each synthesis (e.g. tabulating the study intervention characteristics and comparing against the planned groups for each synthesis (item #5)).	3,4
	13b	Describe any methods required to prepare the data for presentation or synthesis, such as handling of missing summary statistics, or data conversions.	5
	13c	Describe any methods used to tabulate or visually display results of individual studies and syntheses.	5
	13d	Describe any methods used to synthesize results and provide a rationale for the choice(s). If meta-analysis was performed, describe the model(s), method(s) to identify the presence and extent of statistical heterogeneity, and software package(s) used.	4,5
	13e	Describe any methods used to explore possible causes of heterogeneity among study results (e.g. subgroup analysis, meta-regression).	5
	13f	Describe any sensitivity analyses conducted to assess robustness of the synthesized results.	5
Reporting bias assessment	14	Describe any methods used to assess risk of bias due to missing results in a synthesis (arising from reporting biases).	4
Certainty	15	Describe any methods used to assess certainty (or confidence) in the body of evidence for an outcome.	5

For peer review only

For peer review only - http://bmjopen.bmj.com/site/about/guidelines.xhtml

PRISMA 2020 Checklist

Section and Topic	Item #	Checklist item	Location where item is reported
assessment			
**RESULTS**			
Study selection	16a	Describe the results of the search and selection process, from the number of records identified in the search to the number of studies included in the review, ideally using a flow diagram.	
	16b	Cite studies that might appear to meet the inclusion criteria, but which were excluded, and explain why they were excluded.	
Study characteristics	17	Cite each included study and present its characteristics.	
Risk of bias in studies	18	Present assessments of risk of bias for each included study.	
Results of individual studies	19	For all outcomes, present, for each study: (a) summary statistics for each group (where appropriate) and (b) an effect estimate and its precision (e.g. confidence/credible interval), ideally using structured tables or plots.	
Results of syntheses	20a	For each synthesis, briefly summarise the characteristics and risk of bias among contributing studies.	
	20b	Present results of all statistical syntheses conducted. If meta-analysis was done, present for each the summary estimate and its precision (e.g. confidence/credible interval) and measures of statistical heterogeneity. If comparing groups, describe the direction of the effect.	
	20c	Present results of all investigations of possible causes of heterogeneity among study results.	
	20d	Present results of all sensitivity analyses conducted to assess the robustness of the synthesized results.	
Reporting biases	21	Present assessments of risk of bias due to missing results (arising from reporting biases) for each synthesis assessed.	
Certainty of evidence	22	Present assessments of certainty (or confidence) in the body of evidence for each outcome assessed.	
**DISCUSSION**			
Discussion	23a	Provide a general interpretation of the results in the context of other evidence.	
	23b	Discuss any limitations of the evidence included in the review.	
	23c	Discuss any limitations of the review processes used.	6
	23d	Discuss implications of the results for practice, policy, and future research.	6
**OTHER INFORMATION**			
Registration and protocol	24a	Provide registration information for the review, including register name and registration number, or state that the review was not registered.	2
	24b	Indicate where the review protocol can be accessed, or state that a protocol was not prepared.	
	24c	Describe and explain any amendments to information provided at registration or in the protocol.	
Support	25	Describe sources of financial or non-financial support for the review, and the role of the funders or sponsors in the review.	6
Competing interests	26	Declare any competing interests of review authors.	6
Availability of data, code and other materials	27	Report which of the following are publicly available and where they can be found: template data collection forms; data extracted from included studies; data used for all analyses; analytic code; any other materials used in the review.	

For peer review only - http://bmjopen.bmj.com/site/about/guidelines.xhtml

From: Page MJ, McKenzie JE, Bossuyt PM, Boutron I, Hoffmann TC, Mulrow CD, et al. The PRISMA 2020 statement: an updated guideline for reporting systematic reviews. BMJ 2021;372:n71. doi:

PRISMA 2020 Checklist

10.1136/bmj.n71

For more information, visit: http://www.prisma-statement.org/

For peer review only

BMJ Open

The Clinical Utility of Tumor Mutational Burden on Efficacy of Immune Checkpoint Inhibitors in Malignant Solid tumor: Protocol for A Systematic Review and Meta-analysis

Journal:	*BMJ Open*
Manuscript ID	bmjopen-2021-058692.R1
Article Type:	Protocol
Date Submitted by the Author:	14-Jun-2022

Complete List of Authors:	Xiang, Xuemei; People's Liberation Army The General Hospital of Western Theater Command, Basic Medical Laboratory<br>Li, Yunming; People's Liberation Army The General Hospital of Western Theater Command, Department of Information; Southwest Jiaotong University, Department of Statistics<br>Yang, Xiaoguang; People's Liberation Army The General Hospital of Western Theater Command, Department of Information<br>Guo, Wang; Southwest Jiaotong University, Department of Statistics; People's Liberation Army The General Hospital of Western Theater Command, Department of Information<br>Zhou, Pengfei; Southwest Medical University; People's Liberation Army The General Hospital of Western Theater Command, Department of Information

**Primary Subject Heading**:	Oncology
Secondary Subject Heading:	Genetics and genomics, Oncology
Keywords:	Oncogenes < ONCOLOGY, IMMUNOLOGY, ONCOLOGY

SCHOLARONE™
Manuscripts

The Clinical Utility of Tumor Mutational Burden on Efficacy of Immune Checkpoint Inhibitors in Malignant Solid tumor: Protocol for A Systematic Review and Meta-analysis

Xuemei Xiang[1] MS, Yunming Li*[2,3,4] PhD, Xiaoguang Yang[2] MS, Wang Guo[3,2] Mr, Pengfei Zhou[4,2] Mr

1. Basic Medical Laboratory, Medical Support Center, The General Hospital of Western Theater Command，Chengdu, Sichuan Province, 610083, China

2. Department of Information, Medical Support Center, The General Hospital of Western Theater Command, Chengdu, Sichuan Province, 610083, China.

3.Department of Statistics, College of Mathematics, Southwest Jiaotong University, Chengdu, Sichuan Province, 610031, China.

4.School of Public Health, Southwest Medical University, Luzhou, Sichuan Province, 646000, China.

***Corresponding author:** Yunming Li, Male, Ph.D. in epidemiology and health statistics, Postdoctoral in clinical medicine, deputy director technician. Email: lee3082@sina.com, Telephone number: 18908007958. Postal address: 270 Tianhuan Road, Jinniu District, Chengdu, Sichuan, China.

Word count

2066

Keywords: Tumor Mutational Burden; Immune Checkpoint Inhibitors; Solid tumor; Systematic Review

For peer review only

ABSTRACT

Introduction: A major advance in solid malignancies treatment is the development of immune checkpoint inhibitors (ICIs) that have produced durable responses and improved survival. However, the therapeutic effect of ICIs has great heterogeneity in cancer patients. We conduct a systematic review and meta-analysis to evaluate the predictive value of tumor mutation burden (TMB) on efficacy of ICIs.

Methods and analysis: Systematic literature search will be conducted on PubMed, OVID, Web of Science, Embase and the Cochrane Central Register of Controlled Trials Library up to 31 May 2022. The comparison on efficiency of ICIs between TMB high group and TMB low group, which will be measured in terms of odds ratio (OR) of objective response rate/overall response rate (ORR), and hazard ratio (HR) of progression-free survival (PFS) and overall survival (OS). OR of ORR, HR of PFS and OS will be estimated by inverse variance weighted fixed-effects model ($I^2 \leqslant 50\%$) or DerSimonian-Laird random-effects model ($I^2 > 50\%$). In addition, heterogeneity analysis, sensitivity analysis, publication bias and subgroup analysis will be conducted. We plan to conduct a subgroup analysis on age, gender, area, number of patients (High/Low TMB), cancer type, tumor size, stage, line of therapy, TMB sequencing method, type of immunotherapy or follow-up period. Moreover, fractional polynomial regression will be conducted to investigate the dose-response relationship between TMB cutoffs and efficacy of ICIs.

Ethics and dissemination: Ethical approval and informed consent are not required, as the study will be a literature review and will not involve direct contact with patients or alterations to patient care. This systematic review is anticipated to be finished in December 2023, and the results will be published in a peer-reviewed journal.

PROSPERO registration number: CRD42021262480.

Article Summary

Strengths and limitations of this study

► This is an update comprehensive systematic review focused on tumor mutation burden and efficacy of ICIs for the prognosis of patients with solid tumor.

► We plan to conduct a comprehensive subgroup analysis of association between TMB and efficiency of ICIs, including age, gender, area, number of patients (High/Low TMB), tumor size, stage, TMB sequencing method, type of immunotherapy or follow-up period.

► We will focused on the long-term efficacy of ICIs in patients with solid tumor.

► We will search databases of English, while other languages may be ignored.

For peer review only - http://bmjopen.bmj.com/site/about/guidelines.xhtml

Introduction

ICIs have been identified to improve response and survival in diverse solid tumors and hematologic malignancies. However, the efficacy seems satisfactory in some patients, while others do not,[1-6] suggesting eligible biomarkers are required to identify subgroups appropriate for cancer immunotherapy. At present, scientists have recognized several candidate biomarkers, such as programmed cell death ligand 1 (PD-L1), transcriptomic and epigenetic signatures, tumor infiltrating lymphocytes (TILs), oncogenic driver mutations and mismatch repair deficiency (dMMR).[7] Among them, TMB is likely to be a promising biomarker. TMB is broadly defined as the number of somatic mutations per megabase of interrogated genomic sequence. TMB is a continuous variable and variability of TMB (ranging from 0.001/Mb to more than 1000/Mb) has been observed across and within cancer types.[8 9] It was suggested that a higher TMB increase the likelihood of generating immunogenic tumor neoantigens recognized by the host immune system.[10-12]

Retrospective evidence suggests that TMB can predict the efficacy of ICIs, and recent U.S. Food and Drug Administration (FDA) approval of pembrolizumab for the TMB-high tumor subgroup. But, the predict value seems inconsistent in patients with different tumor types, this may be associated with the degree of variability in TMB. Current investigations indicate that some cancer types have less variability in TMB such as lung and head and neck cancers, and some having greater variability such as colon, bladder, and uterine cancers.[13] Studies are attempting to validate the long-term oncologic impact of TMB. Despite a number of studies uncovering powerful forecasting capability of TMB on efficacy of ICIs, however, negative results are also reported, especially in long-term survival.[14-16] Although there are three meta-analyses reporting the predictive value of TMB.[17-19] The sample size of the first two studies is small and the subgroup analysis is incomplete.[17,18] 29 studies were included in the latest one in 2019, with a total of 4431 patients,[19] however, there is also a lack of support of long-term efficacy of all types of tumors due to the insufficient number of studies and patients, and it is not enough to seek out the best threshold of TMB, and studies are far from enough to make a convincing conclusion in small cell lung cancer (SCLC). Moreover, most of the studies done in PD-(L)1 monotherapy, and the research on combined therapy is also insufficient.

Hence, we plan to update comprehensive systematic review and meta-analysis to evaluate the value of tumor mutation burden on efficacy of ICIs in malignant solid tumors, and conduct overall subgroup analyses to identify potential effects of ICIs.

Method

MATERIAL AND METHODS

For peer review only - http://bmjopen.bmj.com/site/about/guidelines.xhtml

We submitted this study protocol to PROSPERO (CRD42021262480). Methods for this systematic review and pairwise meta- analysis follow the Preferred Reporting Items for Systematic Review and Meta- Analysis Protocols.[20 21] As this is a systematic literature research, ethical approval is waived.

Inclusion and exclusion criteria

We will include all prospective or retrospective studies that meet the following criteria:

Population

We will include cohort or clinical trials assessed ICIs, such as PD-1/PD-L1, Cytotoxic T Lymphocyte-associated Antigen-4 (CTLA-4), or their combination, or with chemotherapy, in patients with malignant solid tumors,.

Intervention

ICIs treatment in cancer patients with malignant solid tumors.

Comparator

Efficiency of ICIs therapy will be evaluated by high TMB group and low TMB group. OR of ORR, or HR of PFS or OS, and their 95% confidence intervals (95% CI) were given in the article, or sufficient data is available to calculate them.

Outcome

► Association between TMB and response rate of ICIs in all kinds of malignant solid tumor types, including OS, PFS, DFS, RFS, DSS, et.al.

► Association of subgroup analysis between TMB and efficiency of ICIs,, including age, gender, area, number of patients (High/Low TMB), tumor size, stage, TMB sequencing method, type of immunotherapy or follow-up period.

► Correlations between TMB and clinicopathological features, such as tumor size, stage, and metastasis.

Exclusion criteria:

1. Review, comments, case reports, nonhuman study.
2. The study did not contain a control groups and analysis.
3. The data needed to be extracted in the study is incomplete.

Search strategy

From inception to 31 May 2022, PubMed, Ovid, Web of Science, Embase and the Cochrane Central Register of Controlled Trials will be searched using the MeSH terms “Tumor Burden”, “ Tumor Load”, “ Tumor Weight” and “ Immune Checkpoint Inhibitor”, “ Immune Checkpoint Inhibition”, “ Immune Checkpoint Blockers”, “ Immune Checkpoint Blockade”, “PD-1”, “PD-L1”, “CTLA-4” or the name of the drugs (ie, nivolumab, pembrolizumab, atezolizumab, durvalumab, avelumab, ipilimumab, tremelimumab) and the related keywords “ Mutational Burden” or “ Mutation Burden”.

For peer review only - http://bmjopen.bmj.com/site/about/guidelines.xhtml
For peer review only

The languages will not be limited in our search strategy. The search strategy of Ovid is presented in table 1.

Data abstraction

XMX and WG will independently assess the eligibility of reports from the title and/or abstract. A third reviewer, YML, will join them to resolve any disagreements. Studies that meet the inclusion criteria will be selected for further analysis. For included studies, we will ask for the original data from corresponding authors for diagnosis and prognosis analysis. The following information will be extracted from each study: first author, study design, year of publication, median age, gender, TMB sequencing method, follow-up period, type of cancer, tumor size, stage, type of immunotherapy, TMB cutoff， number of Patients (High/Low TMB) , area of patients, outcomes (ORR, PFS, OS, et.al) and their value. When duplicate publications were identified, the most comprehensive one will be included.

Assessment of risk of bias in included studies and study quality

Two systematic review authors (YML and WG) will independently assess the risk of bias for each study using the Newcastle-Ottawa Scale(NOS). NOS will be adopted to assess the quality of studies included.[22] The total score ranged from 0 to 9, as 8–9 points indicated high quality of a study, five to seven points indicated medium quality, and studies with points lower than five showed poor quality.

Assessment of publication bias

We plan to use the funnel plot and Egger's test to evaluate the potential publication bias by R-4.0.2 , only if at least 10 studies are included. P<0.05 will be considered to indicate significant publication bias.

Assessment of heterogeneity

The χ^2 test will be used to examine heterogeneity in pooling analysis. Heterogeneity is considered to be statistically significant when P <0.10 in these qualitative tests. We plan to use the I^2 test to estimate the proportion of total variation across studies that is attributable to heterogeneity rather than chance, with values of 25%, 50% and 75% indicating low, moderate and high inconsistency, respectively. To determine the source of heterogeneity, we will conduct a meta- regression on different factors within R-4.0.2. We also plan to conduct a subgroup analysis on age, gender, area, number of patients (High/Low TMB), tumor size, stage, TMB sequencing method, type of immunotherapy or follow-up period.

Sensitivity analysis

To determine the robustness of the pooled results, we will conduct a sensitivity analysis by examining individual studies on estimated effects using R-4.0.2.

Data synthesis

The primary endpoint of the meta-analysis are the comparison on efficiency of ICIs between TMB

For peer review only - http://bmjopen.bmj.com/site/about/guidelines.xhtml

high group and TMB low group, which will be measured in terms of OR of ORR, and HR of PFS and OS. Heterogeneity among individual studies will be evaluated by the Q test; I^2> 50% and/or P<0.10 indicated significant heterogeneity.[23] Pooled OR or HR with Z test will be calculated by DerSimonian-Laird random-effects model when significant heterogeneity is identified, otherwise inverse variance weighted fixed-effects model will be adopted. In addition, funnel plots will be constructed, and Begg's test and Egger's test will be performed to evaluate publication bias (P<0.10 is considered to be visible publication bias). Besides, sensitivity analysis will be used to test the stability of the results in the meta-analysis. To further explore variation of effect of TMB on immunotherapy efficiency, subgroup analyses stratified by follow-up period, tumor size, tumor area, stage, line of therapy, TMB sequencing method, type of immunotherapy of ICIs alone (PD-L1, PD-1, CTLA-4 et.al) or ICIs combined with chemo will be conducted. Moreover, to investigate the dose-response relationship between TMB cutoffs and efficacy of ICIs, fractional polynomial regression (two degree) will be conducted on studies of no <50 patients. To note, total mutation burden detected by whole exome sequencing (WES) will be converted to mutations per megabase using a linear transformation.[24] Furthermore, we will evaluate ORR by TMB and PD-L1 expression after layering each other in studies which the two could be both acquired. R-4.0.2 will be used for analyses mentioned above.

Discussion

The rationale for the association between TMB and benefit from immunotherapy is based on the hypothesis that tumor mutation-specific neoantigens can be displayed on major histocompatibility complexes (MHC) on the tumor cell surface, and then recognized by tumor infiltrating T-cells, accordingly, a higher TMB will generate more neoantigens that can then trigger intratumoral T-cells whose ability to attack and destroy tumor cells is enabled by ICIs.[10 12]

As a new biomarker, there is an urgent need to harmonize and standardize TMB measurement, testing platforms and reporting of TMB. Various strategies to optimize TMB as a predictive biomarker of ICIs are being explored. Overall subgroup analysis of patients treated with ICIs will help to evaluate the clinical efficacy for specific cancer types, and it is possible to extend the approval of immunotherapy to a larger patient population.

PATIENT AND PUBLIC INVOLVEMENT

As the study is a protocol of meta- analysis based on previously published literature, the primary patient data will not be collected. Patient or public will not be involved in the study design, recruitment and data analysis.

Contributors: XMX, WG and YML conceived the study and drafted the manuscript. XMX and YML registered the protocol systematic review in the PROSPERO database. YML and XGY designed the search strategy. PFZ, WG and YML formed the data synthesis and analysis plan. XMX, XGY and YML supervised this study and revised the manuscript.

For peer review only - http://bmjopen.bmj.com/site/about/guidelines.xhtml

Founding: Military Medical Research Project, the General Hospital of Western Theater Command, Chinese People's Liberation Army (2019ZY10, 2021-XZYG-A14); Special Scientific Research Project of Army Health Care (21BJZ39); 2021 Basic Research Cultivation Project of the Central Universities (2682021ZTPY018)

Competing interests of Reviewer: None.

Patient consent for publication: Not required.

Provenance and peer review: Not commissioned; externally peer reviewed.

References

1. Schadendorf D, Hodi FS, Robert C, et al. Pooled Analysis of Long-Term Survival Data From Phase II and Phase III Trials of Ipilimumab in Unresectable or Metastatic Melanoma. *Journal of clinical oncology : official journal of the American Society of Clinical Oncology* 2015;33(17):1889-94. doi: 10.1200/jco.2014.56.2736 [published Online First: 2015/02/11]
2. Overman MJ, Lonardi S, Wong KYM, et al. Durable Clinical Benefit With Nivolumab Plus Ipilimumab in DNA Mismatch Repair-Deficient/Microsatellite Instability-High Metastatic Colorectal Cancer. *Journal of clinical oncology : official journal of the American Society of Clinical Oncology* 2018;36(8):773-79. doi: 10.1200/jco.2017.76.9901 [published Online First: 2018/01/23]
3. Motzer RJ, Tannir NM, McDermott DF, et al. Nivolumab plus Ipilimumab versus Sunitinib in Advanced Renal-Cell Carcinoma. *The New England journal of medicine* 2018;378(14):1277-90. doi: 10.1056/NEJMoa1712126 [published Online First: 2018/03/22]
4. Rosenberg JE, Hoffman-Censits J, Powles T, et al. Atezolizumab in patients with locally advanced and metastatic urothelial carcinoma who have progressed following treatment with platinum-based chemotherapy: a single-arm, multicentre, phase 2 trial. *Lancet (London, England)* 2016;387(10031):1909-20. doi: 10.1016/s0140-6736(16)00561-4 [published Online First: 2016/03/10]
5. Wolchok JD, Kluger H, Callahan MK, et al. Nivolumab plus ipilimumab in advanced melanoma. *The New England journal of medicine* 2013;369(2):122-33. doi: 10.1056/NEJMoa1302369 [published Online First: 2013/06/04]
6. Hodi FS, O'Day SJ, McDermott DF, et al. Improved survival with ipilimumab in patients with metastatic melanoma. *The New England journal of medicine* 2010;363(8):711-23. doi: 10.1056/NEJMoa1003466 [published Online First: 2010/06/08]
7. Havel JJ, Chowell D, Chan TA. The evolving landscape of biomarkers for checkpoint inhibitor immunotherapy. *Nature reviews Cancer* 2019;19(3):133-50. doi: 10.1038/s41568-019-0116-x [published Online First: 2019/02/14]
8. Alexandrov LB, Nik-Zainal S, Wedge DC, et al. Signatures of mutational processes in human cancer. *Nature* 2013;500(7463):415-21. doi: 10.1038/nature12477 [published Online First: 2013/08/16]
9. Chalmers ZR, Connelly CF, Fabrizio D, et al. Analysis of 100,000 human cancer genomes reveals the landscape of tumor mutational burden. *Genome medicine*

For peer review only

2017;9(1):34. doi: 10.1186/s13073-017-0424-2 [published Online First: 2017/04/20]
10. Chan TA, Wolchok JD, Snyder A. Genetic Basis for Clinical Response to CTLA-4 Blockade in Melanoma. *The New England journal of medicine* 2015;373(20):1984. doi: 10.1056/NEJMc1508163 [published Online First: 2015/11/13]
11. Van Allen EM, Miao D, Schilling B, et al. Genomic correlates of response to CTLA-4 blockade in metastatic melanoma. *Science (New York, NY)* 2015;350(6257):207-11. doi: 10.1126/science.aad0095 [published Online First: 2015/09/12]
12. Rizvi NA, Hellmann MD, Snyder A, et al. Cancer immunology. Mutational landscape determines sensitivity to PD-1 blockade in non-small cell lung cancer. *Science (New York, NY)* 2015;348(6230):124-8. doi: 10.1126/science.aaa1348 [published Online First: 2015/03/15]
13. Merino DM, McShane LM, Fabrizio D, et al. Establishing guidelines to harmonize tumor mutational burden (TMB): in silico assessment of variation in TMB quantification across diagnostic platforms: phase I of the Friends of Cancer Research TMB Harmonization Project. *Journal for immunotherapy of cancer* 2020;8(1) doi: 10.1136/jitc-2019-000147 [published Online First: 2020/03/29]
14. Tang B, Yan X, Sheng X, et al. Safety and clinical activity with an anti-PD-1 antibody JS001 in advanced melanoma or urologic cancer patients. *Journal of hematology & oncology* 2019;12(1):7. doi: 10.1186/s13045-018-0693-2 [published Online First: 2019/01/16]
15. Morrison C, Pabla S, Conroy JM, et al. Predicting response to checkpoint inhibitors in melanoma beyond PD-L1 and mutational burden. *Journal for immunotherapy of cancer* 2018;6(1):32. doi: 10.1186/s40425-018-0344-8 [published Online First: 2018/05/11]
16. Mishima S, Kawazoe A, Nakamura Y, et al. Clinicopathological and molecular features of responders to nivolumab for patients with advanced gastric cancer. *Journal for immunotherapy of cancer* 2019;7(1):24. doi: 10.1186/s40425-019-0514-3 [published Online First: 2019/02/02]
17. Kim JY, Kronbichler A, Eisenhut M, et al. Tumor Mutational Burden and Efficacy of Immune Checkpoint Inhibitors: A Systematic Review and Meta-Analysis. *Cancers* 2019;11(11) doi: 10.3390/cancers11111798 [published Online First: 2019/11/17]
18. Wu Y, Xu J, Du C, et al. The Predictive Value of Tumor Mutation Burden on Efficacy of Immune Checkpoint Inhibitors in Cancers: A Systematic Review and Meta-Analysis. *Frontiers in oncology* 2019;9:1161. doi: 10.3389/fonc.2019.01161 [published Online First: 2019/11/22]
19. Zhu J, Zhang T, Li J, et al. Association Between Tumor Mutation Burden (TMB) and Outcomes of Cancer Patients Treated With PD-1/PD-L1 Inhibitions: A Meta-Analysis. *Frontiers in pharmacology* 2019;10:673. doi: 10.3389/fphar.2019.00673 [published Online First: 2019/07/02]
20. Moher D, Shamseer L, Clarke M, et al. Preferred reporting items for systematic review and meta-analysis protocols (PRISMA-P) 2015 statement. *Systematic reviews* 2015;4(1):1. doi: 10.1186/2046-4053-4-1 [published Online First: 2015/01/03]
21. Shamseer L, Moher D, Clarke M, et al. Preferred reporting items for systematic

For peer review only

For peer review only - http://bmjopen.bmj.com/site/about/guidelines.xhtml

review and meta-analysis protocols (PRISMA-P) 2015: elaboration and explanation. *BMJ (Clinical research ed)* 2015;350:g7647. doi: 10.1136/bmj.g7647 [published Online First: 2015/01/04]

22. Stang A. Critical evaluation of the Newcastle-Ottawa scale for the assessment of the quality of nonrandomized studies in meta-analyses. *European journal of epidemiology* 2010;25(9):603-5. doi: 10.1007/s10654-010-9491-z [published Online First: 2010/07/24]
23. Higgins JP, Thompson SG, Deeks JJ, et al. Measuring inconsistency in meta-analyses. *BMJ (Clinical research ed)* 2003;327(7414):557-60. doi: 10.1136/bmj.327.7414.557 [published Online First: 2003/09/06]
24. Chang H, Sasson A, Srinivasan S, et al. Bioinformatic Methods and Bridging of Assay Results for Reliable Tumor Mutational Burden Assessment in Non-Small-Cell Lung Cancer. *Molecular diagnosis & therapy* 2019;23(4):507-20. doi: 10.1007/s40291-019-00408-y [published Online First: 2019/06/30]

For peer review only

Table 1. Search strategy (OVID)

Item	Search strategy
1	Immune Checkpoint Inhibitor/ or Immune Checkpoint Inhibition/or Immune Checkpoint Blockers / or Immune Checkpoint Blockade / or PD-1 / or PD-L1/ or CTLA-4/ or nivolumab/ or pembrolizumab / or atezolizumab / or avelumab / or durvalumab/ or tremelimumab / or ipilimumab /
2	mutation/ or mutational/ or burden/ or weight.mp.
3	1 and 2
4	tumor/ or cancer/ or neoplasms.mp.
5	3 and 4

For peer review only

Abbreviations:

ICIs= Immune Checkpoint Inhibitors

TMB = Tumor Mutation Burden

OR = Odds Ratio

ORR = Objective Response Rate /Overall Response Rate (ORR)

HR = Hazard Ratio

OS = Overall Survival

PFS = Progression-free Survival

DFS = Disease-free Survival

RFS = Recurrence-free Survival

DSS = Disease-specific Survival

SCLC = Small Cell Lung Cancer

FDA = U.S. Food and Drug Administration

CTLA-4 = Cytotoxic T Lymphocyte-associated Antigen-4

dMMR = Oncogenic Driver Mutations and Mismatch Repair Deficiency

PD-L1 = Programmed Cell Death Ligand 1

PD-1 = Programmed Cell Death 1

TILs = Tumorinfiltrating Lymphocytes

CI = Confidence Interval

NOS = Newcastle-Ottawa Scale

WES = Whole Exome Sequencing

MHC = Major Histocompatibility Complexes

RCT = Randomized Controlled Trial.

For peer review only

PRISMA 2020 Checklist

Section and Topic	Item #	Checklist item	Location where item is reported
**TITLE**			
Title	1	Identify the report as a systematic review.	1
**ABSTRACT**			
Abstract	2	See the PRISMA 2020 for Abstracts checklist.	2
**INTRODUCTION**			
Rationale	3	Describe the rationale for the review in the context of existing knowledge.	3
Objectives	4	Provide an explicit statement of the objective(s) or question(s) the review addresses.	3
**METHODS**			
Eligibility criteria	5	Specify the inclusion and exclusion criteria for the review and how studies were grouped for the syntheses.	3,4
Information sources	6	Specify all databases, registers, websites, organisations, reference lists and other sources searched or consulted to identify studies. Specify the date when each source was last searched or consulted.	4
Search strategy	7	Present the full search strategies for all databases, registers and websites, including any filters and limits used.	4
Selection process	8	Specify the methods used to decide whether a study met the inclusion criteria of the review, including how many reviewers screened each record and each report retrieved, whether they worked independently, and if applicable, details of automation tools used in the process.	4
Data collection process	9	Specify the methods used to collect data from reports, including how many reviewers collected data from each report, whether they worked independently, any processes for obtaining or confirming data from study investigators, and if applicable, details of automation tools used in the process.	4
Data items	10a	List and define all outcomes for which data were sought. Specify whether all results that were compatible with each outcome domain in each study were sought (e.g. for all measures, time points, analyses), and if not, the methods used to decide which results to collect.	3,4
	10b	List and define all other variables for which data were sought (e.g. participant and intervention characteristics, funding sources). Describe any assumptions made about any missing or unclear information.	3,4
Study risk of bias assessment	11	Specify the methods used to assess risk of bias in the included studies, including details of the tool(s) used, how many reviewers assessed each study and whether they worked independently, and if applicable, details of automation tools used in the process.	4
Effect measures	12	Specify for each outcome the effect measure(s) (e.g. risk ratio, mean difference) used in the synthesis or presentation of results.	3,4
Synthesis methods	13a	Describe the processes used to decide which studies were eligible for each synthesis (e.g. tabulating the study intervention characteristics and comparing against the planned groups for each synthesis (item #5)).	3,4
	13b	Describe any methods required to prepare the data for presentation or synthesis, such as handling of missing summary statistics, or data conversions.	5
	13c	Describe any methods used to tabulate or visually display results of individual studies and syntheses.	5
	13d	Describe any methods used to synthesize results and provide a rationale for the choice(s). If meta-analysis was performed, describe the model(s), method(s) to identify the presence and extent of statistical heterogeneity, and software package(s) used.	4,5
	13e	Describe any methods used to explore possible causes of heterogeneity among study results (e.g. subgroup analysis, meta-regression).	5
	13f	Describe any sensitivity analyses conducted to assess robustness of the synthesized results.	5
Reporting bias assessment	14	Describe any methods used to assess risk of bias due to missing results in a synthesis (arising from reporting biases).	4
Certainty	15	Describe any methods used to assess certainty (or confidence) in the body of evidence for an outcome.	5

For peer review only - http://bmjopen.bmj.com/site/about/guidelines.xhtml

PRISMA 2020 Checklist

Section and Topic	Item #	Checklist item	Location where item is reported
assessment			
**RESULTS**			
Study selection	16a	Describe the results of the search and selection process, from the number of records identified in the search to the number of studies included in the review, ideally using a flow diagram.	
	16b	Cite studies that might appear to meet the inclusion criteria, but which were excluded, and explain why they were excluded.	
Study characteristics	17	Cite each included study and present its characteristics.	
Risk of bias in studies	18	Present assessments of risk of bias for each included study.	
Results of individual studies	19	For all outcomes, present, for each study: (a) summary statistics for each group (where appropriate) and (b) an effect estimate and its precision (e.g. confidence/credible interval), ideally using structured tables or plots.	
Results of syntheses	20a	For each synthesis, briefly summarise the characteristics and risk of bias among contributing studies.	
	20b	Present results of all statistical syntheses conducted. If meta-analysis was done, present for each the summary estimate and its precision (e.g. confidence/credible interval) and measures of statistical heterogeneity. If comparing groups, describe the direction of the effect.	
	20c	Present results of all investigations of possible causes of heterogeneity among study results.	
	20d	Present results of all sensitivity analyses conducted to assess the robustness of the synthesized results.	
Reporting biases	21	Present assessments of risk of bias due to missing results (arising from reporting biases) for each synthesis assessed.	
Certainty of evidence	22	Present assessments of certainty (or confidence) in the body of evidence for each outcome assessed.	
**DISCUSSION**			
Discussion	23a	Provide a general interpretation of the results in the context of other evidence.	
	23b	Discuss any limitations of the evidence included in the review.	
	23c	Discuss any limitations of the review processes used.	6
	23d	Discuss implications of the results for practice, policy, and future research.	6
**OTHER INFORMATION**			
Registration and protocol	24a	Provide registration information for the review, including register name and registration number, or state that the review was not registered.	2
	24b	Indicate where the review protocol can be accessed, or state that a protocol was not prepared.	
	24c	Describe and explain any amendments to information provided at registration or in the protocol.	
Support	25	Describe sources of financial or non-financial support for the review, and the role of the funders or sponsors in the review.	6
Competing interests	26	Declare any competing interests of review authors.	6
Availability of data, code and other materials	27	Report which of the following are publicly available and where they can be found: template data collection forms; data extracted from included studies; data used for all analyses; analytic code; any other materials used in the review.	

For peer review only

From: Page MJ, McKenzie JE, Bossuyt PM, Boutron I, Hoffmann TC, Mulrow CD, et al. The PRISMA 2020 statement: an updated guideline for reporting systematic reviews. BMJ 2021;372:n71. doi:

PRISMA 2020 Checklist

10.1136/bmj.n71

For more information, visit: http://www.prisma-statement.org/

For peer review only

The Clinical Utility of Tumour Mutational Burden on Efficacy of Immune Checkpoint Inhibitors in Malignant Solid Tumour: Protocol for A Systematic Review and Meta-analysis

Journal:	*BMJ Open*
Manuscript ID	bmjopen-2021-058692.R2
Article Type:	Protocol
Date Submitted by the Author:	04-Jul-2022
Complete List of Authors:	Xiang, Xuemei; People's Liberation Army The General Hospital of Western Theater Command, Basic Medical Laboratory<br>Li, Yunming; People's Liberation Army The General Hospital of Western Theater Command, Department of Information; Southwest Jiaotong University, Department of Statistics<br>Yang, Xiaoguang; People's Liberation Army The General Hospital of Western Theater Command, Department of Information<br>Guo, Wang; Southwest Jiaotong University, Department of Statistics; People's Liberation Army The General Hospital of Western Theater Command, Department of Information<br>Zhou, Pengfei; Southwest Medical University; People's Liberation Army The General Hospital of Western Theater Command, Department of Information
**Primary Subject Heading**:	Oncology
Secondary Subject Heading:	Genetics and genomics, Oncology
Keywords:	Oncogenes < ONCOLOGY, IMMUNOLOGY, ONCOLOGY

The Clinical Utility of Tumour Mutational Burden on Efficacy of Immune Checkpoint Inhibitors in Malignant Solid Tumours: Protocol for A Systematic Review and Meta-analysis

Xuemei Xiang[1] MS, Yunming Li*[2,3,4] PhD, Xiaoguang Yang[2] MS, Wang Guo[3,2] Mr, Pengfei Zhou[4,2] Mr

1. Basic Medical Laboratory, Medical Support Center, The General Hospital of Western Theater Command，Chengdu, Sichuan Province, 610083, China
2. Department of Information, Medical Support Center, The General Hospital of Western Theater Command, Chengdu, Sichuan Province, 610083, China.
3.Department of Statistics, College of Mathematics, Southwest Jiaotong University, Chengdu, Sichuan Province, 610031, China.
4.School of Public Health, Southwest Medical University, Luzhou, Sichuan Province, 646000, China.

***Corresponding author:** Yunming Li, Male, Ph.D. in epidemiology and health statistics, Postdoctoral in clinical medicine, deputy director technician. Email: lee3082@sina.com, Telephone number: 18908007958. Postal address: 270 Tianhuan Road, Jinniu District, Chengdu, Sichuan, China.

Word count
2174
Keywords: Tumor Mutational Burden; Immune Checkpoint Inhibitors; Solid tumor; Systematic Review

For peer review only

ABSTRACT

Introduction: A major advance in solid malignancy treatment is the development of immune checkpoint inhibitors (ICIs), which have produced durable responses and increased survival rates. However, the therapeutic effect of ICIs has great heterogeneity in cancer patients. We propose a systematic review and meta-analysis to evaluate the predictive value of tumour mutation burden (TMB) on efficacy of ICIs.

Methods and analysis: A systematic literature search will be conducted in the PubMed, OVID, Web of Science, Embase and Cochrane Central Register of Controlled Trials Library databases up to 31 May 2022. The comparison of the efficacy of ICIs between TMB high group and TMB low group will be measured in terms of the odds ratio (OR) of the objective response rate/overall response rate (ORR), and the hazard ratio (HRs) of progression-free survival (PFS) and overall survival (OS). The OR of ORR, and the HRs of PFS and OS will be estimated by an inverse variance weighted fixed-effects model ($I^2 \leqslant 50\%$) or a DerSimonian–Laird random-effects model ($I^2 > 50\%$). In addition, heterogeneity analysis, sensitivity analysis, publication bias and subgroup analysis will be conducted. We plan to conduct a subgroup analysis on age, sex, area, number of patients (high/low TMB), cancer type, tumour size, stage, line of therapy, TMB sequencing method, type of immunotherapy and follow-up period. Moreover, fractional polynomial regression will be conducted to investigate the dose–response relationship between TMB cut-off and the efficacy of ICIs.

Ethics and dissemination: Ethical approval and informed consent are not needed, as the study will be a literature review and will not involve direct contact with patients or alterations to patient care. This systematic review is anticipated to be finished in December 2023, and the results will be published in a peer-reviewed journal.

PROSPERO registration number: CRD42021262480.

Article Summary

Strengths and limitations of this study

► This will be an update comprehensive systematic review focused on the tumour mutation burden and the efficacy of ICIs for the prognosis of patients with solid tumours.

► We plan to conduct a comprehensive subgroup analysis of the association between TMB and the efficacy of ICIs, including age, sex, area, number of patients (high/low TMB), tumour size, stage, TMB sequencing method, type of immunotherapy and follow-up period.

► We will focus on the long-term efficacy of ICIs in patients with solid tumours.

► We will search databases for studies published in English, while other languages may be ignored.

Introduction

For peer review only

For peer review only - http://bmjopen.bmj.com/site/about/guidelines.xhtml

ICIs have been shown to improve response and increase survival rates in diverse solid tumours and haematologic malignancies. However, the efficacy of ICIs seems satisfactory in some patientsand unsatisfactory in others,[1-6] suggesting the need to identify biomarkers that indicate which subgroups are candidates for cancer immunotherapy. At present, scientists have recognized several potential biomarkers, such as programmed cell death ligand 1 (PD-L1), transcriptomic and epigenetic signatures, tumour infiltrating lymphocytes (TILs), oncogenic driver mutations and mismatch repair deficiency (dMMR).[7] Among them, TMB is likely to be a promising biomarker. TMB is broadly defined as the number of somatic mutations per megabase of interrogated genomic sequence. TMB is a continuous variable and variability of TMB (ranging from 0.001/Mb to more than 1000/Mb) has been observed across and within cancer types.[8 9]It was suggested that a higher TMB increases the likelihood of generating immunogenic tumour neoantigens recognized by the host immune system.[10-12]

Retrospective evidence suggests that TMB can predict the efficacy of ICIs, and recent U.S. Food and Drug Administration (FDA) approval of pembrolizumab for the TMB-high tumour subgroup. However, the predictive value seems inconsistent in patients with different tumour types, which may be associated with the degree of variability in TMB. Current investigations indicate that some cancer types have less variability in TMB such as lung and head and neck cancers, and some have greater variability such as colon, bladder, and uterine cancers.[13] Studies are attempting to validate the long-term oncologic impact of TMB. Despite a number of studies uncovering the powerful forecasting capability of TMB on the efficacy of the ICIsnegative results have also been reported, especially in long-term survival.[14-16] However, there are three meta-analyses reporting the predictive value of TMB.[17-19] The sample size of the first two studies was small and the subgroup analysis was incomplete.[17,18] Twenty-nine studies were included in the latest meta-analysis from in 2019, with a total of 4431 patients,[19] However, there is also a lack of evidence regarding the long-term efficacy of all types of tumours due to the insufficient number of studies and patients. It is not sufficient to seek out the best threshold for TMB, and there is no consensus regarding the use of this biomarker for in small cell lung cancer (SCLC). Moreover, most of the studies were performed in PD-(L)1 monotherapy, and the research on combined therapy is also insufficient.

Hence, we propose an update to the evidence by conducting a comprehensive systematic review and meta-analysis to evaluate the value of tumour mutation burden on the efficacy of ICIs in malignant solid tumours . We will also conduct overall subgroup analyses to identify the potential effects of ICIs.

Method

Materials and methods

For peer review only - http://bmjopen.bmj.com/site/about/guidelines.xhtml

We submitted this study protocol to PROSPERO (CRD42021262480). This systematic review and pairwise meta- analysis will be conducted in accordance with the Preferred Reporting Items for Systematic Review and Meta- Analysis Protocols.[20 21] As this is a systematic literature study, ethical approval was waived.

Inclusion and exclusion criteria

We will include all prospective or retrospective studies that meet the following criteria:

Population

We will include cohort or clinical trials assessing ICIs, such as PD-1/PD-L1, cytotoxic T lymphocyte--associated antigen-4 (CTLA-4), or their combination, or with chemotherapy, in patients with malignant solid tumours. A cut-off of ⩾ 10 mutations per megabase (mut/Mb) was chosen to define the "high TMB" patient population.

Intervention

ICI treatment in cancer patients with malignant solid tumours.

Comparator

The efficacy of ICI therapy will be evaluated in the high TMB group and the low TMB group. The OR of ORR or the HR of PFS or OS, and their 95% confidence intervals (95% CI) are given in the article, or sufficient data are available to calculate them.

Outcome

► Association between TMB and response rate of ICIs in all kinds of malignant solid tumor types, including OS, PFS, DFS, RFS, DSS, et.al.

► Association of subgroup analysis between TMB and efficacy of ICIs,, including age, sex, area, number of patients (high/low TMB), tumour size, stage, TMB sequencing method, type of immunotherapy or follow-up period.

► Correlations between TMB and clinicopathological features, such as tumour size, stage, and metastasis.

The exclusion criteria will be as follows:

1. Review, comments, case reports, nonhuman study.
2. The study does not contain a control groups and analysis.
3. The data are incomplete.

Search strategy

The PubMed, Ovid, Web of Science, Embase and the Cochrane Central Register of Controlled Trials databases will be searched from inception to 31 May 2022, using the MeSH terms " Immune Checkpoint Inhibitors" and the related keywords " Immune Checkpoint Inhibition", " Immune Checkpoint Blockers", " Immune Checkpoint Blockade", "PD-1", "PD-L1", "CTLA-4" or the name of the drugs (i.e.,nivolumab, pembrolizumab, atezolizumab, durvalumab, avelumab, ipilimumab,

For peer review only

tremelimumab) , “ Mutational Burden” or “ Mutation Burden”. The languages will not be limited in our search strategy. The search strategy for Ovid is presented in Table 1 and the full search strategies and the results of 5 databases are presented in supplementary files 1

Data abstraction

XMX and WG will independently assess the eligibility of reports from the title and/or abstract. A third reviewer, YML, will be consulted in case of disagreements. Studies that meet the inclusion criteria will be selected for further analysis. For the included studies, we will ask for the original data from corresponding authors for diagnosis and prognosis analysis. The following information will be extracted from each study: first author, study design, year of publication, median age, sex, TMB sequencing method, follow-up period, type of cancer, tumour size, stage, type of immunotherapy, TMB cut-off, number of patients (high/low TMB), ,area of patients, and outcomes (ORR, PFS, OS, etc.) . When duplicate publications are identified, the most comprehensive study will beincluded.

Assessment of risk of bias in included studies and study quality

Two systematic review authors (YML and WG) will independently assess the risk of bias for each study using the Newcastle-Ottawa Scale(NOS). The NOS will be adopted to assess the quality of the included studies.[22] The total score ranges from 0 to 9, where 8–9 points indicates high quality of a study, five to seven points indicated medium quality, and less than five points indicates poor quality.

Assessment of publication bias

If at least 10 studies are included, we plan to use the funnel plot and Egger’s test to evaluate the potential publication bias by R-4.0.2. P<0.05 will be considered to indicate significant publication bias.

Assessment of heterogeneity

The χ^2 test will be used to examine heterogeneity in pooling analysis. Heterogeneity is considered to be statistically significant when P <0.10 in these qualitative tests. We plan to use the I^2 test to estimate the proportion of total variation across studies that is attributable to heterogeneity rather than chance, with values of 25%, 50% and 75% indicating low, moderate and high heterogeneity, respectively. To determine the source of heterogeneity, we will conduct a meta- regression on different factors within R-4.0.2. We also plan to conduct a subgroup analysis on age, sex, area, number of patients (high/low TMB), tumour size, stage, TMB sequencing method, type of immunotherapy or follow-up period.

Sensitivity analysis

To determine the robustness of the pooled results, we will conduct a sensitivity analysis by examining individual studies on estimated effects using R-4.0.2.

Data synthesis

The primary endpoint of the meta-analysis is the comparison of the efficacy of ICIs between the TMB high group and TMB low group, which will be measured in terms of the OR of ORR and the

For peer review only - http://bmjopen.bmj.com/site/about/guidelines.xhtml

HRs of PFS and OS. Heterogeneity among individual studies will be evaluated by the Q test; I^2> 50% and/or P<0.10 will be considered to indicate significant heterogeneity.[23] Pooled ORs or HRs with Z test will be calculated by DerSimonian-Laird random-effects model when significant heterogeneity is identified, otherwise, inverse variance weighted fixed-effects model will be adopted. In addition, funnel plots will be constructed, and Begg's test and Egger's test will be performed to evaluate publication bias (P<0.10 is considered to indicate visible publication bias). In addition, sensitivity analysis will be used to test the stability of the results in the meta-analysis. To further explore the variation in the effect of TMB on immunotherapy efficacy, subgroup analyses stratified by follow-up period, tumour size, tumour area, stage, line of therapy, TMB sequencing method, type of immunotherapy of ICIs alone (PD-L1, PD-1, CTLA-4 , etc.) or ICIs combined with chemotherapy will be conducted. Moreover, to investigate the dose–response relationship between TMB cut-off and the efficacy of ICIs, fractional polynomial regression (two degrees) will be conducted on studies with at least 50patients. Notably, the total mutation burden detected by whole exome sequencing (WES) will be converted to mutations per megabase using linear transformation.[24] Furthermore, we will evaluate the ORR by TMB and PD-L1 expression after layering in studies in which the two could be both acquired. R-4.0.2 will be used for the analyses mentioned above.

PATIENT AND PUBLIC INVOLVEMENT

As the study is a protocol of meta- analysis based on previously published literature, the primary patient data will not be collected. Patients or the public will not be involved in the study design, recruitment or data analysis.

Discussion

The rationale for the association between TMB and benefit from immunotherapy is based on the hypothesis that tumour mutation-specific neoantigens can be displayed on major histocompatibility complexes (MHC) on the tumour cell surface, and then recognized by tumour infiltrating Tcells, accordingly, a higher TMB will generate more neoantigens that can then trigger intratumoral Tcells whose ability to attack and destroy tumour cells is enabled by ICIs.[10 12]

As a new biomarker, there is an urgent need to harmonize and standardize TMB measurement, testing platforms and reporting of TMB. Various strategies to optimize TMB as a predictive biomarker of ICIs are being explored. Overall subgroup analysis of patients treated with ICIs will help to evaluate the clinical efficacy for specific cancer types, and it is possible to extend the approval of immunotherapy to a larger patient population.

Ethics and Dissemination

The data included in this project will be collected from the original studies; therefore, ethical approval and informed consent of patients will not be needed.

Contributors: XMX, WG and YML conceived the study and drafted the manuscript. XMX and YML

For peer review only - http://bmjopen.bmj.com/site/about/guidelines.xhtml

registered the protocol systematic review in the PROSPERO database. YML and XGY designed the search strategy. PFZ, WG and YML formed the data synthesis and analysis plan. XMX, XGY and YML supervised this study and revised the manuscript.

Funding: Military Medical Research Project, the General Hospital of Western Theater Command, Chinese People's Liberation Army (2019ZY10, 2021-XZYG-A14); Special Scientific Research Project of Army Health Care (21BJZ39); 2021 Basic Research Cultivation Project of the Central Universities (2682021ZTPY018)

Competing interests: None declared.

Patient consent for publication: Not needed..

Provenance and peer review: Not commissioned; externally peer reviewed.

Data availability statement Data are available upon reasonable request

For peer review only

References

1. Schadendorf D, Hodi FS, Robert C, et al. Pooled Analysis of Long-Term Survival Data From Phase II and Phase III Trials of Ipilimumab in Unresectable or Metastatic Melanoma. *Journal of clinical oncology : official journal of the American Society of Clinical Oncology* 2015;33(17):1889-94. doi: 10.1200/jco.2014.56.2736 [published Online First: 2015/02/11]
2. Overman MJ, Lonardi S, Wong KYM, et al. Durable Clinical Benefit With Nivolumab Plus Ipilimumab in DNA Mismatch Repair-Deficient/Microsatellite Instability-High Metastatic Colorectal Cancer. *Journal of clinical oncology : official journal of the American Society of Clinical Oncology* 2018;36(8):773-79. doi: 10.1200/jco.2017.76.9901 [published Online First: 2018/01/23]
3. Motzer RJ, Tannir NM, McDermott DF, et al. Nivolumab plus Ipilimumab versus Sunitinib in Advanced Renal-Cell Carcinoma. *The New England journal of medicine* 2018;378(14):1277-90. doi: 10.1056/NEJMoa1712126 [published Online First: 2018/03/22]
4. Rosenberg JE, Hoffman-Censits J, Powles T, et al. Atezolizumab in patients with locally advanced and metastatic urothelial carcinoma who have progressed following treatment with platinum-based chemotherapy: a single-arm, multicentre, phase 2 trial. *Lancet (London, England)* 2016;387(10031):1909-20. doi: 10.1016/s0140-6736(16)00561-4 [published Online First: 2016/03/10]
5. Wolchok JD, Kluger H, Callahan MK, et al. Nivolumab plus ipilimumab in advanced melanoma. *The New England journal of medicine* 2013;369(2):122-33. doi: 10.1056/NEJMoa1302369 [published Online First: 2013/06/04]
6. Hodi FS, O'Day SJ, McDermott DF, et al. Improved survival with ipilimumab in patients with metastatic melanoma. *The New England journal of medicine* 2010;363(8):711-23. doi: 10.1056/NEJMoa1003466 [published Online First: 2010/06/08]
7. Havel JJ, Chowell D, Chan TA. The evolving landscape of biomarkers for checkpoint inhibitor immunotherapy. *Nature reviews Cancer* 2019;19(3):133-50. doi: 10.1038/s41568-019-0116-x [published Online First: 2019/02/14]

For peer review only - http://bmjopen.bmj.com/site/about/guidelines.xhtml

8. Alexandrov LB, Nik-Zainal S, Wedge DC, et al. Signatures of mutational processes in human cancer. *Nature* 2013;500(7463):415-21. doi: 10.1038/nature12477 [published Online First: 2013/08/16]
9. Chalmers ZR, Connelly CF, Fabrizio D, et al. Analysis of 100,000 human cancer genomes reveals the landscape of tumor mutational burden. *Genome medicine* 2017;9(1):34. doi: 10.1186/s13073-017-0424-2 [published Online First: 2017/04/20]
10. Chan TA, Wolchok JD, Snyder A. Genetic Basis for Clinical Response to CTLA-4 Blockade in Melanoma. *The New England journal of medicine* 2015;373(20):1984. doi: 10.1056/NEJMc1508163 [published Online First: 2015/11/13]
11. Van Allen EM, Miao D, Schilling B, et al. Genomic correlates of response to CTLA-4 blockade in metastatic melanoma. *Science (New York, NY)* 2015;350(6257):207-11. doi: 10.1126/science.aad0095 [published Online First: 2015/09/12]
12. Rizvi NA, Hellmann MD, Snyder A, et al. Cancer immunology. Mutational landscape determines sensitivity to PD-1 blockade in non-small cell lung cancer. *Science (New York, NY)* 2015;348(6230):124-8. doi: 10.1126/science.aaa1348 [published Online First: 2015/03/15]
13. Merino DM, McShane LM, Fabrizio D, et al. Establishing guidelines to harmonize tumor mutational burden (TMB): in silico assessment of variation in TMB quantification across diagnostic platforms: phase I of the Friends of Cancer Research TMB Harmonization Project. *Journal for immunotherapy of cancer* 2020;8(1) doi: 10.1136/jitc-2019-000147 [published Online First: 2020/03/29]
14. Tang B, Yan X, Sheng X, et al. Safety and clinical activity with an anti-PD-1 antibody JS001 in advanced melanoma or urologic cancer patients. *Journal of hematology & oncology* 2019;12(1):7. doi: 10.1186/s13045-018-0693-2 [published Online First: 2019/01/16]
15. Morrison C, Pabla S, Conroy JM, et al. Predicting response to checkpoint inhibitors in melanoma beyond PD-L1 and mutational burden. *Journal for immunotherapy of cancer* 2018;6(1):32. doi: 10.1186/s40425-018-0344-8 [published Online First: 2018/05/11]
16. Mishima S, Kawazoe A, Nakamura Y, et al. Clinicopathological and molecular features of responders to nivolumab for patients with advanced gastric cancer. *Journal for immunotherapy of cancer* 2019;7(1):24. doi: 10.1186/s40425-019-0514-3 [published Online First: 2019/02/02]
17. Kim JY, Kronbichler A, Eisenhut M, et al. Tumor Mutational Burden and Efficacy of Immune Checkpoint Inhibitors: A Systematic Review and Meta-Analysis. *Cancers* 2019;11(11) doi: 10.3390/cancers11111798 [published Online First: 2019/11/17]
18. Wu Y, Xu J, Du C, et al. The Predictive Value of Tumor Mutation Burden on Efficacy of Immune Checkpoint Inhibitors in Cancers: A Systematic Review and Meta-Analysis. *Frontiers in oncology* 2019;9:1161. doi: 10.3389/fonc.2019.01161 [published Online First: 2019/11/22]
19. Zhu J, Zhang T, Li J, et al. Association Between Tumor Mutation Burden (TMB) and Outcomes of Cancer Patients Treated With PD-1/PD-L1 Inhibitions: A Meta-Analysis. *Frontiers in pharmacology* 2019;10:673. doi: 10.3389/fphar.2019.00673 [published Online First: 2019/07/02]

For peer review only

20. Moher D, Shamseer L, Clarke M, et al. Preferred reporting items for systematic review and meta-analysis protocols (PRISMA-P) 2015 statement. *Systematic reviews* 2015;4(1):1. doi: 10.1186/2046-4053-4-1 [published Online First: 2015/01/03]
21. Shamseer L, Moher D, Clarke M, et al. Preferred reporting items for systematic review and meta-analysis protocols (PRISMA-P) 2015: elaboration and explanation. *BMJ (Clinical research ed)* 2015;350:g7647. doi: 10.1136/bmj.g7647 [published Online First: 2015/01/04]
22. Stang A. Critical evaluation of the Newcastle-Ottawa scale for the assessment of the quality of nonrandomized studies in meta-analyses. *European journal of epidemiology* 2010;25(9):603-5. doi: 10.1007/s10654-010-9491-z [published Online First: 2010/07/24]
23. Higgins JP, Thompson SG, Deeks JJ, et al. Measuring inconsistency in meta-analyses. *BMJ (Clinical research ed)* 2003;327(7414):557-60. doi: 10.1136/bmj.327.7414.557 [published Online First: 2003/09/06]
24. Chang H, Sasson A, Srinivasan S, et al. Bioinformatic Methods and Bridging of Assay Results for Reliable Tumor Mutational Burden Assessment in Non-Small-Cell Lung Cancer. *Molecular diagnosis & therapy* 2019;23(4):507-20. doi: 10.1007/s40291-019-00408-y [published Online First: 2019/06/30]

For peer review only

For peer review only - http://bmjopen.bmj.com/site/about/guidelines.xhtml

Table 1. Search strategy (OVID)

Item	Search strategy
#1	exp Immune Checkpoint Inhibitors/
#2	((immunotherap*) or (immune checkpoint inhibit*) or (ICI) or (immune checkpoint inhibit*) or (ICIs) or (immune checkpoint block*) or (ICB) or (ICBs) or (pembrolizumab) or (avelumab) or (nivolumab) or (durvalumab) or (tremelimumab) or (atezolizumab) or (Ipilimumab) or (Cemiplimab) or (tiragolumab) or (Dostarlimab) or (Camrelizumab) or (PD-1) or (programmed death 1) or (PD-L1) or (programmed death-ligand 1) or (anti-PD-1) or (anti-PD-L1) or (CTLA-4) or (Cytotoxic T-lymphocyte antigen 4)).tw.
#3	#1 OR #2
#4	((Carcinoma) or (Neoplasms) or (Cancer) or (Tumour) or (Tumor)).tw.
#5	#3 and #4
#6	((mutation burden) or (mutational burden) or (mutation load) or (mutational load) or (TMB) or (TML)).tw.
#7	#5 and #6

For peer review only - http://bmjopen.bmj.com/site/about/guidelines.xhtml
For peer review only

Abbreviations:

ICIs= Immune Checkpoint Inhibitors

TMB = Tumor Mutation Burden

OR = Odds Ratio

ORR = Objective Response Rate /Overall Response Rate (ORR)

HR = Hazard Ratio

OS = Overall Survival

PFS = Progression-free Survival

DFS = Disease-free Survival

RFS = Recurrence-free Survival

DSS = Disease-specific Survival

SCLC = Small Cell Lung Cancer

FDA = U.S. Food and Drug Administration

CTLA-4 = Cytotoxic T Lymphocyte-associated Antigen-4

dMMR = Oncogenic Driver Mutations and Mismatch Repair Deficiency

PD-L1 = Programmed Cell Death Ligand 1

PD-1 = Programmed Cell Death 1

TILs = Tumorinfiltrating Lymphocytes

CI = Confidence Interval

NOS = Newcastle-Ottawa Scale

WES = Whole Exome Sequencing

MHC = Major Histocompatibility Complexes

RCT = Randomized Controlled Trial.

For peer review only

For peer review only - http://bmjopen.bmj.com/site/about/guidelines.xhtml

Search strategies and results of 5 databases

Database: PubMed, OVID, Embase, the Cochrane Central Register of Controlled Trials Library databases, Web of Science.

Data Run: 01/07/2022

database	Search strategies	results
Pubmed	#1 "Immune Checkpoint Inhibitors" [Mesh Terms]	6109
	#2 "immunotherap*"[Title/Abstract] OR "immune checkpoint inhibit*"[Title/Abstract] OR "ICI"[Title/Abstract] OR "ICIs"[Title/Abstract] OR "immune checkpoint block*"[Title/Abstract] OR "ICB"[Title/Abstract] OR "ICBs"[Title/Abstract] OR "pembrolizumab"[Title/Abstract] OR "avelumab"[Title/Abstract] OR "nivolumab"[Title/Abstract] OR "durvalumab"[Title/Abstract] OR "tremelimumab"[Title/Abstract] OR "atezolizumab"[Title/Abstract] OR "Ipilimumab"[Title/Abstract] OR "Cemiplimab"[Title/Abstract] OR "tiragolumab"[Title/Abstract] OR "Dostarlimab*"[Title/Abstract] OR "Camrelizumab"[Title/Abstract] OR "PD-1"[Title/Abstract] OR "programmed death 1"[Title/Abstract] OR "PD-L1"[Title/Abstract] OR "programmed death-ligand 1"[Title/Abstract] OR "PD-1/PD-L1"[Title/Abstract] OR "anti–PD-1/anti–PD-L1"[Title/Abstract] OR "CTLA-4"[Title/Abstract] OR "Cytotoxic T-lymphocyte antigen 4"[Title/Abstract]	164405
	#3 #1 OR #2	164676
	#4 "Carcinoma"[Title/Abstract] OR "Neoplasms"[Title/Abstract] OR "Cancer"[Title/Abstract] OR "Tumour" [Title/Abstract] OR "Tumor"[Title/Abstract]	3285875
	#5 #3 and #4	102540
	#6 "mutation burden"[Title/Abstract] OR "mutational burden"[Title/Abstract] OR "mutation load"[Title/Abstract] OR "mutational load"[Title/Abstract] OR "TMB"[Title/Abstract] OR "TML"[Title/Abstract]	10061
	#7 #5 and #6	3268
OVID	#1 exp Immune Checkpoint Inhibitors/	14572
	#2 ((immunotherap*) or (immune checkpoint inhibit*) or (ICI) or (immune checkpoint inhibit*) or (ICIs) or (immune checkpoint block*) or (ICB) or (ICBs) or (pembrolizumab) or (avelumab) or (nivolumab) or (durvalumab) or (tremelimumab) or (atezolizumab) or (Ipilimumab) or (Cemiplimab) or (tiragolumab) or (Dostarlimab) or (Camrelizumab) or (PD-1) or (programmed death 1) or (PD-L1) or (programmed death-ligand 1) or (anti-PD-1) or (anti-PD-L1) or (CTLA-4) or (Cytotoxic T-lymphocyte antigen 4)).tw.	153834
	#3 #1 OR #2	156787
	#4 ((Carcinoma) or (Neoplasms) or (Cancer) or (Tumour) or (Tumor)).tw.	3136257
	#5 #3 and #4	94825
	#6 ((mutation burden) or (mutational burden) or (mutation load) or (mutational load) or (TMB) or (TML)).tw.	9467
	#7 #5 and #6	2963
Embase	#1 'immunotherap*:ab,ti OR 'immune checkpoint inhibit*':ab,ti OR 'ici':ab,ti OR 'icis':ab,ti OR 'immune checkpoint block*':ab,ti OR 'icb':ab,ti OR 'icbs':ab,ti OR 'pembrolizumab':ab,ti OR 'avelumab':ab,ti OR 'nivolumab':ab,ti OR 'durvalumab':ab,ti OR 'tremelimumab':ab,ti OR 'atezolizumab':ab,ti OR 'ipilimumab':ab,ti OR 'cemiplimab':ab,ti OR 'tiragolumab':ab,ti OR 'dostarlimab*':ab,ti OR 'camrelizumab':ab,ti OR 'pd-1':ab,ti OR 'programmed death 1':ab,ti OR 'pd-l1':ab,ti OR 'programmed death-ligand 1':ab,ti OR 'pd-1/pd-l1':ab,ti OR 'anti–pd-1/anti–pd-l1':ab,ti OR 'ctla-4':ab,ti OR 'cytotoxic t-lymphocyte atigen 4':ab,ti	251032
	#2 'Carcinoma':ab,ti OR 'Neoplasms':ab,ti OR 'Cancer':ab,ti OR 'Tumour' ab,ti OR 'Tumor':ab,ti	1809315
	#3 #1 and #2	101253
	#4 'mutation burden':ab,ti OR 'mutational burden':ab,ti OR 'mutation load':ab,ti OR 'mutational load':ab,ti OR 'TMB':ab,ti OR 'TML':ab,ti	16290
	#5 #3 and #4	5654
Cochrane	#1 ("immunotherap* OR immune checkpoint inhibit* OR ICI OR ICIs OR immune checkpoint block* OR ICB OR ICBs OR pembrolizumab OR avelumab OR nivolumab OR durvalumab OR tremelimumab OR atezolizumab OR Ipilimumab OR Cemiplimab OR tiragolumab OR Dostarlimab* OR Camrelizumab	21080

For peer review only

	OR PD-1 OR programmed death 1 OR PD-L1 OR programmed death-ligand 1 OR PD-1/PD-L1 OR anti–PD-1/anti–PD-L1 ORCTLA-4 OR Cytotoxic T-lymphocyte antigen 4):ti,ab,kw<br>#2 (Carcinoma OR Neoplasms OR Cancer OR Tumour OR Tumor):ti,ab,kw<br>#3 #1 and #2<br>#4 (mutation burden OR mutational burden OR mutation load OR mutational load OR TMB OR TML):ti,ab,kw<br>#5 #3 and #4	 <br>242648<br>13396<br>1495<br><br>460
web of science	#1 (TI=(“immunotherap*” OR “immune checkpoint inhibit*” OR “ICI” OR "ICIs" OR “immune checkpoint block*” OR "ICB" OR "ICBs" OR “pembrolizumab” OR “avelumab” OR “nivolumab” OR “durvalumab” OR “tremelimumab” OR “atezolizumab” OR “Ipilimumab” OR “Cemiplimab” OR “tiragolumab” OR “Dostarlimab*” OR “Camrelizumab” OR “PD-1” OR “programmed death 1” OR “PD-L1” OR “programmed death-ligand 1” OR “PD-1/PD-L1” OR “anti–PD-1/anti–PD-L1” OR “CTLA-4” OR “Cytotoxic T-lymphocyte atigen 4”)) OR AB=(“immunotherap*” OR “immune checkpoint inhibit*” OR “ICI” OR "ICIs" OR “immune checkpoint block*” OR "ICB" OR "ICBs" OR “pembrolizumab” OR “avelumab” OR “nivolumab” OR “durvalumab” OR “tremelimumab” OR “atezolizumab” OR “Ipilimumab” OR “Cemiplimab” OR “tiragolumab” OR “Dostarlimab*” OR “Camrelizumab” OR “PD-1” OR “programmed death 1” OR “PD-L1” OR “programmed death-ligand 1” OR “PD-1/PD-L1” OR “anti–PD-1/anti–PD-L1” OR “CTLA-4” OR “Cytotoxic T-lymphocyte antigen 4”)<br>#2 (TS = ("Carcinoma" OR " Neoplasms" OR " Cancer" OR " Tumour" OR " Tumor")) OR AB = ("Carcinoma" OR " Neoplasms" OR " Cancer" OR " Tumour" OR " Tumor")<br>#3 #1 and #2<br>#4 (TS = ("mutation burden" OR "mutational burden" OR "mutation load" OR "mutational load" OR "TMB" OR "TML")) OR AB = ("mutation burden" OR "mutational burden" OR "mutation load" OR "mutational load" OR "TMB" OR "TML")<br>#5 #3 and #4	191683<br><br>4045332<br><br>111505<br>13058<br><br>3510

For peer review only

PRISMA-P (Preferred Reporting Items for Systematic review and Meta-Analysis Protocols) 2015 checklist: recommended items to address in a systematic review protocol*

Section and topic	Item No	Checklist item	Location where item is reported
**ADMINISTRATIVE INFORMATION**			
Title:			
Identification	1a	Identify the report as a protocol of a systematic review	1
Update	1b	If the protocol is for an update of a previous systematic review, identify as such	1
Registration	2	If registered, provide the name of the registry (such as PROSPERO) and registration number	2
Authors:			
Contact	3a	Provide name, institutional affiliation, e-mail address of all protocol authors; provide physical mailing address of corresponding author	1
Contributions	3b	Describe contributions of protocol authors and identify the guarantor of the review	7
Amendments	4	If the protocol represents an amendment of a previously completed or published protocol, identify as such and list changes; otherwise, state plan for documenting important protocol amendments	3
Support:			
Sources	5a	Indicate sources of financial or other support for the review	7
Sponsor	5b	Provide name for the review funder and/or sponsor	7
Role of sponsor or funder	5c	Describe roles of funder(s), sponsor(s), and/or institution(s), if any, in developing the protocol	None
**INTRODUCTION**			
Rationale	6	Describe the rationale for the review in the context of what is already known	3
Objectives	7	Provide an explicit statement of the question(s) the review will address with reference to participants, interventions, comparators, and outcomes (PICO)	3,4
**METHODS**			
Eligibility criteria	8	Specify the study characteristics (such as PICO, study design, setting, time frame) and report characteristics (such as years considered, language, publication status) to be used as criteria for eligibility for the review	4
Information sources	9	Describe all intended information sources (such as electronic databases, contact with study authors, trial registers or other grey literature sources) with planned dates of coverage	4,5
Search strategy	10	Present draft of search strategy to be used for at least one electronic database, including planned limits, such that it could be repeated	10

For peer review only

Study records:			
Data management	11a	Describe the mechanism(s) that will be used to manage records and data throughout the review	5
Selection process	11b	State the process that will be used for selecting studies (such as two independent reviewers) through each phase of the review (that is, screening, eligibility and inclusion in meta-analysis)	5
Data collection process	11c	Describe planned method of extracting data from reports (such as piloting forms, done independently, in duplicate), any processes for obtaining and confirming data from investigators	5
Data items	12	List and define all variables for which data will be sought (such as PICO items, funding sources), any pre-planned data assumptions and simplifications	5
Outcomes and prioritization	13	List and define all outcomes for which data will be sought, including prioritization of main and additional outcomes, with rationale	4
Risk of bias in individual studies	14	Describe anticipated methods for assessing risk of bias of individual studies, including whether this will be done at the outcome or study level, or both; state how this information will be used in data synthesis	5
Data synthesis	15a	Describe criteria under which study data will be quantitatively synthesised	5
	15b	If data are appropriate for quantitative synthesis, describe planned summary measures, methods of handling data and methods of combining data from studies, including any planned exploration of consistency (such as I^2, Kendall's τ)	5,6
	15c	Describe any proposed additional analyses (such as sensitivity or subgroup analyses, meta-regression)	5,6
	15d	If quantitative synthesis is not appropriate, describe the type of summary planned	None
Meta-bias(es)	16	Specify any planned assessment of meta-bias(es) (such as publication bias across studies, selective reporting within studies)	5
Confidence in cumulative evidence	17	Describe how the strength of the body of evidence will be assessed (such as GRADE)	None

*** It is strongly recommended that this checklist be read in conjunction with the PRISMA-P Explanation and Elaboration (cite when available) for important clarification on the items. Amendments to a review protocol should be tracked and dated.** **The copyright for PRISMA-P (including checklist) is held by the PRISMA-P Group and is distributed under a Creative Commons Attribution Licence 4.0.**

From: Shamseer L, Moher D, Clarke M, Ghersi D, Liberati A, Petticrew M, Shekelle P, Stewart L, PRISMA-P Group. Preferred reporting items for systematic review and meta-analysis protocols (PRISMA-P) 2015: elaboration and explanation. BMJ. 2015 Jan 2;349(jan02 1):g7647.

BMJ Open

The Clinical Utility of Tumour Mutational Burden on Efficacy of Immune Checkpoint Inhibitors in Malignant Solid Tumour: Protocol for A Systematic Review and Meta-analysis

Journal:	*BMJ Open*
Manuscript ID	bmjopen-2021-058692.R3
Article Type:	Protocol
Date Submitted by the Author:	06-Jul-2022
Complete List of Authors:	Xiang, Xuemei; People's Liberation Army The General Hospital of Western Theater Command, Basic Medical Laboratory<br>Li, Yunming; People's Liberation Army The General Hospital of Western Theater Command, Department of Information; Southwest Jiaotong University, Department of Statistics<br>Yang, Xiaoguang; People's Liberation Army The General Hospital of Western Theater Command, Department of Information<br>Guo, Wang; Southwest Jiaotong University, Department of Statistics; People's Liberation Army The General Hospital of Western Theater Command, Department of Information<br>Zhou, Pengfei; Southwest Medical University; People's Liberation Army The General Hospital of Western Theater Command, Department of Information
**Primary Subject Heading**:	Oncology
Secondary Subject Heading:	Genetics and genomics, Oncology
Keywords:	Oncogenes < ONCOLOGY, IMMUNOLOGY, ONCOLOGY

SCHOLARONE™
Manuscripts

The Clinical Utility of Tumour Mutational Burden on Efficacy of Immune Checkpoint Inhibitors in Malignant Solid Tumours: Protocol for A Systematic Review and Meta-analysis

Xuemei Xiang[1] MS, Yunming Li*[2,3,4] PhD, Xiaoguang Yang[2] MS, Wang Guo[3,2] Mr, Pengfei Zhou[4,2] Mr

1. Basic Medical Laboratory, Medical Support Center, The General Hospital of Western Theater Command，Chengdu, Sichuan Province, 610083, China

2. Department of Information, Medical Support Center, The General Hospital of Western Theater Command, Chengdu, Sichuan Province, 610083, China.

3.Department of Statistics, College of Mathematics, Southwest Jiaotong University, Chengdu, Sichuan Province, 610031, China.

4.School of Public Health, Southwest Medical University, Luzhou, Sichuan Province, 646000, China.

***Corresponding author:** Yunming Li, Male, Ph.D. in epidemiology and health statistics, Postdoctoral in clinical medicine, deputy director technician. Email: lee3082@sina.com, Telephone number: 18908007958. Postal address: 270 Tianhuan Road, Jinniu District, Chengdu, Sichuan, China.

Word count

1990

Keywords: Tumor Mutational Burden; Immune Checkpoint Inhibitors; Solid tumor; Systematic Review

For peer review only

ABSTRACT

Introduction: A major development in solid malignancy treatment is the application of immune checkpoint inhibitors (ICIs), which have produced durable responses and increased survival rates. However, the therapeutic effect of ICIs has great heterogeneity in cancer patients. We propose a systematic review to evaluate the predictive value of tumour mutation burden (TMB) on efficacy of ICIs.

Methods and analysis: A systematic literature search will be conducted in the PubMed, OVID, Web of Science, Embase and Cochrane Central Register of Controlled Trials Library databases up to 31 May 2022. We will compare the efficacy of ICIs between TMB high group and TMB low group in terms of the hazard ratio (HRs) of overall survival (OS) and progression-free survival (PFS), and the odds ratio (OR) of the objective response rate/overall response rate (ORR), . The HRs of PFS and OS,and the OR of ORR, will be measured by an inverse variance weighted fixed-effects model ($I^2 \leqslant$ 50%) or a DerSimonian–Laird random-effects model ($I^2>$ 50%). In addition, subgroup analysis, sensitivity analysis, heterogeneity analysis and publication bias will be conducted. We plan to conduct a subgroup analysis on age, sex, area, number of patients (high/low TMB), cancer type, tumour size, stage, line of therapy, TMB sequencing method, type of immunotherapy and follow-up period.

Ethics and dissemination: Ethical approval and informed consent are not needed, as the study will be a literature review and will not involve direct contact with patients or alterations to patient care. This systematic review is anticipated to be finished in December 2023, and the results will be published in a peer-reviewed journal.

PROSPERO registration number: CRD42021262480.

Article Summary

Strengths and limitations of this study

► This will be an update comprehensive systematic review focused on the tumour mutation burden and the efficacy of ICIs for the prognosis of patients with solid tumours.

► We plan to conduct a comprehensive subgroup analysis of the association between TMB and the efficacy of ICIs, including age, sex, area, number of patients (high/low TMB), tumour size, stage, TMB sequencing method, type of immunotherapy and follow-up period.

► We will focus on the long-term efficacy of ICIs in patients with solid tumours.

► We will search databases for studies published in English, while other languages may be ignored.

Introduction

ICIs have been shown to prolong response and increase survival rates in various solid tumours and haematologic malignancies. However, the efficacy of ICIs seems satisfactory in some patients and unsatisfactory in others,[1-6] suggesting the need to identify biomarkers that indicate which subgroups

For peer review only - http://bmjopen.bmj.com/site/about/guidelines.xhtml

are candidates for malignancy immunotherapy. Nowadays, researchers have identified several potential biomarkers, such as tumour infiltrating lymphocytes (TILs) and programmed cell death ligand 1 (PD-L1, transcriptomic epigenetic signatures and oncogenic driver mutations.[7] Among them, TMB is likely to be a potential biomarker. TMB is broadly defined as the number of somatic mutations per megabase of interrogated genomic sequence[8]. TMB is a continuous variable and variability of TMB (ranging from 0.001/Mb to more than 1000/Mb) has been observed across and within cancer types.[9,10]It was suggested that a higher TMB increases the likelihood of generating immunogenic tumour neoantigens recognized by the host immune system.[11-13]

Retrospective evidence suggests that TMB can predict the efficacy of ICIs, and recent U.S. Food and Drug Administration (FDA) approval of pembrolizumab for the TMB-high tumour subgroup. However, the predictive value seems inconsistent in patients with different tumour types, which may be associated with the degree of variability in TMB. Current investigations indicate that some cancer types have less variability in TMB such as lung and head and neck cancers, and some have greater variability such as colon, bladder, and uterine cancers.[14] Studies are attempting to validate the long-term oncologic impact of TMB. Although numerous studies have revealed the exciting forecasting capability of TMB on the efficacy of the ICIs,negative results have also been reported, especially in long-term survival.[15-17] As far as we know, three meta-analyses reported the predictive value of TMB.[18-20] The sample size of the first two studies was small and the subgroup analysis was incomplete.[17,18] The latest meta-analysis published in 2019 including twenty-nine studies, with a total of 4431 patients,[19] However, there is also a lack of evidence regarding the long-term efficacy of all types of tumours due to the insufficient number of studies and patients. It is not sufficient to seek out the best threshold for TMB, and there is no consensus regarding the use of this biomarker for in small cell lung cancer (SCLC). Moreover, in most studies PD-(L)1 monotherapy were performed, and the research on combined therapy is also insufficient.

Hence, we propose an update to the evidence by conducting a comprehensive systematic review and meta-analysis to evaluate the value of TMB on the efficacy of ICIs in malignant solid tumours . We will also proceed overall subgroup analyses to determine the promising effects of ICIs.

Method

Materials and methods

We submitted this study protocol to PROSPERO (CRD42021262480). This systematic review and meta- analysis will be conducted in accordance with the Preferred Reporting Items for Systematic Review and Meta- Analysis Protocols.[21,22]

Inclusion and exclusion criteria

We will include all prospective or retrospective studies that meet the following criteria:

Population

For peer review only - http://bmjopen.bmj.com/site/about/guidelines.xhtml

We will include cohort or clinical trials assessing ICIs, such as PD-1/PD-L1, cytotoxic T lymphocyte--associated antigen-4 (CTLA-4), or their combination, or with chemotherapy, in patients with malignant solid tumours. A cut-off of ⩾ 10 mutations per megabase (mut/Mb) was chosen to define the “high TMB” patient population.

Intervention

ICI treatment in cancer patients with malignant solid tumours.

Comparator

We will evaluate the efficacy of ICI therapy in the TMB high group and the TMB low group. The HRs of PFS, the HRs of OS, the OR of ORR, and their 95% confidence intervals (95% CI) are reported in our article. Besides, we will calculate them using the sufficient data collected in studies.

Outcome

► Association between different levels of TMB and response rate of ICIs in all kinds of malignant solid tumor types, including OS, PFS, DFS, RFS, DSS, et.al.

► Association of subgroup analysis between different levels of TMB and efficacy of ICIs,, including age, sex, area, number of patients (high/low TMB), tumour size, stage, TMB sequencing method, type of immunotherapy or follow-up period.

► Correlations between TMB and clinicopathological features, such as tumour size, stage, and metastasis.

The exclusion criteria will be as follows:

1. Review, comments, case reports, nonhuman study.
2. There is no control groups and analysis.
3. The data are incomplete.

Search strategy

The PubMed, Ovid, Web of Science, Embase and the Cochrane Central Register of Controlled Trials databases will be searched from inception to 31 May 2022, using the MeSH terms “ Immune Checkpoint Inhibitors” and the related keywords “ Immune Checkpoint Inhibition”, “ Immune Checkpoint Blockers”, “ Immune Checkpoint Blockade”, “PD-L1”, “CTLA-4”, “PD-1”or the name of the drugs (i.e.,atezolizumab, pembrolizumab, nivolumab, durvalumab, ipilimumab, avelumab, tremelimumab) , “ Mutational Burden” or “ Mutation Burden”. The languages will not be limited in our search strategy. The search strategy for Ovid is presented in Table 1 and the full search strategies and the results of 5 databases are presented in supplementary files 1

Data abstraction

XMX and WG will independently assess the eligibility of reports from the title and/or abstract. A third reviewer, YML, will be consulted in case of inconsistent. We will select studies that meet the inclusion criteria for further analysis. For the included studies that have no insufficient data, we will

For peer review only - http://bmjopen.bmj.com/site/about/guidelines.xhtml

ask for the original data from corresponding authors analysis. The following items will be extracted from all included studies: first author, study design, year of publication, median age, sex, TMB sequencing method, follow-up period, type of cancer, tumour size, stage, type of immunotherapy, TMB cut-off, number of patients (high/low TMB), ,area of patients, and outcomes (PFS, ORR, OS, etc.) ..

Assessment of risk of bias in included studies and study quality

Two systematic review authors (YML and WG) will independently assess the risk of bias for each study using the Newcastle-Ottawa Scale(NOS). The NOS will be adopted to assess the quality of the included studies.[23] The total score ranges from 0 to 9, where 8–9 points indicates high quality of a study, five to seven points indicated medium quality, and less than five points indicates poor quality.

Assessment of publication bias

If at least 10 studies are included, we plan to use Egger's test and the funnel plot to estimate the potential publication bias by R-4.0.2. $P<0.05$ will be considered to indicate significant publication bias.

Assessment of heterogeneity

The χ^2 test will be used to estimate heterogeneity in pooling analysis. Heterogeneity is considered to be statistically significant when $P<0.10$ in all qualitative tests. The I^2 test will be used to examine the proportion of total variation, with values of 25%, 50% and 75% indicating low, moderate and high heterogeneity, respectively. We plan to conduct a meta- regression to confirm the source of heterogeneity within R-4.0.2. We also plan to conduct a subgroup analysis on age, sex, area, number of patients (high/low TMB), tumour size, stage, TMB sequencing method, type of immunotherapy or follow-up period.

Sensitivity analysis

To determine the robustness of the pooled results, sensitivity analysis will be performed by examining individual studies using R-4.0.2.

Data synthesis

The primary outcome of this article is the comparison of the efficacy of ICIs between the TMB high group and TMB low group, which will be assessed by the HRs of PFS and OS ,and the OR of ORR d. Heterogeneity among individual studies will be evaluated by the Q test; $I^2> 50\%$ and/or $P<0.10$ will be considered to indicate significant heterogeneity.[24] DerSimonian-Laird random-effects model will be used to calculate the pooled ORs or HRs with Z test when significant heterogeneity is identified. Otherwise, inverse variance weighted fixed-effects model will be adopted. In addition, To evaluate publication bias, the Begg's test and Egger's test will be applied. Funnel plots will be constructed. In addition, the stability of the results in our article will be tested by sensitivity analysis. To further explore the variation in the effect of TMB on immunotherapy efficacy, subgroup analyses stratified by follow-up period, tumour size, tumour area, stage, line of therapy, TMB sequencing

For peer review only - http://bmjopen.bmj.com/site/about/guidelines.xhtml

method, type of immunotherapy of ICIs alone (PD-L1, PD-1, CTLA-4 , etc.) or ICIs combined with chemotherapy will be conducted. Notably, theTMB detected by whole exome sequencing (WES) will be converted to mutations per megabase using linear transformation.[25] R-4.0.2 will be used for the analyses mentioned above.

Patient and Public Involvement

As our study is a protocol of meta-analysis, which based on previously published literature, the primary patient data will not need to be collected. Existing databases will be used for the purpose of this study. The public or patients will not be involved in the study design, recruitment or data analysis.

Ethics and Dissemination

The data included in this project will be collected from the original studies; therefore, ethical approval and informed consent of patients will not be needed. This systematic review will assess the predict value of TMB in patients with malignant solid tumours. Patients treated with ICIs with high/low levels of TMB will eventually benefit from the knowledge of this study. We will publish the results of this protocol as a complete meta-review paper in an academic journal and scientific conferences.

Contributors: XMX, WG and YML conceived the study and drafted the manuscript. XMX and YML registered the protocol systematic review in the PROSPERO database. YML and XGY designed the search strategy. PFZ, WG and YML formed the data synthesis and analysis plan. XMX, XGY and YML supervised this study and revised the manuscript.

Funding: Military Medical Research Project, the General Hospital of Western Theater Command, Chinese People's Liberation Army (2019ZY10, 2021-XZYG-A14); Special Scientific Research Project of Army Health Care (21BJZ39); 2021 Basic Research Cultivation Project of the Central Universities (2682021ZTPY018)**Competing interests:** None declared.

Patient consent for publication: Not needed..

Provenance and peer review: Not commissioned; externally peer reviewed.

Data availability statement Data are available upon reasonable request.

For peer review only

References

1. Schadendorf D, Hodi FS, Robert C, et al. Pooled Analysis of Long-Term Survival Data From Phase II and Phase III Trials of Ipilimumab in Unresectable or Metastatic Melanoma. *Journal of clinical oncology : official journal of the American Society of Clinical Oncology.* 2015;33(17):1889-1894.
2. Overman MJ, Lonardi S, Wong KYM, et al. Durable Clinical Benefit With Nivolumab Plus Ipilimumab in DNA Mismatch Repair-Deficient/Microsatellite Instability-High Metastatic Colorectal Cancer. *Journal of clinical oncology : official journal of the American Society of Clinical Oncology.* 2018;36(8):773-779.
3. Motzer RJ, Tannir NM, McDermott DF, et al. Nivolumab plus Ipilimumab versus Sunitinib in Advanced Renal-Cell Carcinoma. *The New England journal of*

For peer review only - http://bmjopen.bmj.com/site/about/guidelines.xhtml

medicine. 2018;378(14):1277-1290.
4. Rosenberg JE, Hoffman-Censits J, Powles T, et al. Atezolizumab in patients with locally advanced and metastatic urothelial carcinoma who have progressed following treatment with platinum-based chemotherapy: a single-arm, multicentre, phase 2 trial. *Lancet (London, England).* 2016;387(10031):1909-1920.
5. Wolchok JD, Kluger H, Callahan MK, et al. Nivolumab plus ipilimumab in advanced melanoma. *The New England journal of medicine.* 2013;369(2):122-133.
6. Hodi FS, O'Day SJ, McDermott DF, et al. Improved survival with ipilimumab in patients with metastatic melanoma. *The New England journal of medicine.* 2010;363(8):711-723.
7. Havel JJ, Chowell D, Chan TA. The evolving landscape of biomarkers for checkpoint inhibitor immunotherapy. *Nature reviews. Cancer.* 2019;19(3):133-150.
8. Ning B, Liu Y, Wang M, Li Y, Xu T, Wei Y. The Predictive Value of Tumor Mutation Burden on Clinical Efficacy of Immune Checkpoint Inhibitors in Melanoma: A Systematic Review and Meta-Analysis. *Frontiers in pharmacology.* 2022;13:748674.
9. Alexandrov LB, Nik-Zainal S, Wedge DC, et al. Signatures of mutational processes in human cancer. *Nature.* 2013;500(7463):415-421.
10. Chalmers ZR, Connelly CF, Fabrizio D, et al. Analysis of 100,000 human cancer genomes reveals the landscape of tumor mutational burden. *Genome medicine.* 2017;9(1):34.
11. Chan TA, Wolchok JD, Snyder A. Genetic Basis for Clinical Response to CTLA-4 Blockade in Melanoma. *The New England journal of medicine.* 2015;373(20):1984.
12. Van Allen EM, Miao D, Schilling B, et al. Genomic correlates of response to CTLA-4 blockade in metastatic melanoma. *Science (New York, N.Y.).* 2015;350(6257):207-211.
13. Rizvi NA, Hellmann MD, Snyder A, et al. Cancer immunology. Mutational landscape determines sensitivity to PD-1 blockade in non-small cell lung cancer. *Science (New York, N.Y.).* 2015;348(6230):124-128.
14. Merino DM, McShane LM, Fabrizio D, et al. Establishing guidelines to harmonize tumor mutational burden (TMB): in silico assessment of variation in TMB quantification across diagnostic platforms: phase I of the Friends of Cancer Research TMB Harmonization Project. *Journal for immunotherapy of cancer.* 2020;8(1).
15. Tang B, Yan X, Sheng X, et al. Safety and clinical activity with an anti-PD-1 antibody JS001 in advanced melanoma or urologic cancer patients. *Journal of hematology & oncology.* 2019;12(1):7.
16. Morrison C, Pabla S, Conroy JM, et al. Predicting response to checkpoint inhibitors in melanoma beyond PD-L1 and mutational burden. *Journal for immunotherapy of cancer.* 2018;6(1):32.
17. Mishima S, Kawazoe A, Nakamura Y, et al. Clinicopathological and molecular features of responders to nivolumab for patients with advanced gastric cancer.

For peer review only - http://bmjopen.bmj.com/site/about/guidelines.xhtml

Journal for immunotherapy of cancer. 2019;7(1):24.
18. Kim JY, Kronbichler A, Eisenhut M, et al. Tumor Mutational Burden and Efficacy of Immune Checkpoint Inhibitors: A Systematic Review and Meta-Analysis. *Cancers.* 2019;11(11).
19. Wu Y, Xu J, Du C, et al. The Predictive Value of Tumor Mutation Burden on Efficacy of Immune Checkpoint Inhibitors in Cancers: A Systematic Review and Meta-Analysis. *Frontiers in oncology.* 2019;9:1161.
20. Zhu J, Zhang T, Li J, et al. Association Between Tumor Mutation Burden (TMB) and Outcomes of Cancer Patients Treated With PD-1/PD-L1 Inhibitions: A Meta-Analysis. *Frontiers in pharmacology.* 2019;10:673.
21. Moher D, Shamseer L, Clarke M, et al. Preferred reporting items for systematic review and meta-analysis protocols (PRISMA-P) 2015 statement. *Systematic reviews.* 2015;4(1):1.
22. Shamseer L, Moher D, Clarke M, et al. Preferred reporting items for systematic review and meta-analysis protocols (PRISMA-P) 2015: elaboration and explanation. *BMJ (Clinical research ed.).* 2015;350:g7647.
23. Stang A. Critical evaluation of the Newcastle-Ottawa scale for the assessment of the quality of nonrandomized studies in meta-analyses. *European journal of epidemiology.* 2010;25(9):603-605.
24. Higgins JP, Thompson SG, Deeks JJ, Altman DG. Measuring inconsistency in meta-analyses. *BMJ (Clinical research ed.).* 2003;327(7414):557-560.
25. Chang H, Sasson A, Srinivasan S, et al. Bioinformatic Methods and Bridging of Assay Results for Reliable Tumor Mutational Burden Assessment in Non-Small-Cell Lung Cancer. *Molecular diagnosis & therapy.* 2019;23(4):507-520.

For peer review only

Table 1. Search strategy (OVID)

For peer review only - http://bmjopen.bmj.com/site/about/guidelines.xhtml

Item	Search strategy
#1	exp Immune Checkpoint Inhibitors/
#2	((immunotherap*) or (immune checkpoint inhibit*) or (ICI) or (immune checkpoint inhibit*) or (ICIs) or (immune checkpoint block*) or (ICB) or (ICBs) or (pembrolizumab) or (avelumab) or (nivolumab) or (durvalumab) or (tremelimumab) or (atezolizumab) or (Ipilimumab) or (Cemiplimab) or (tiragolumab) or (Dostarlimab) or (Camrelizumab) or (PD-1) or (programmed death 1) or (PD-L1) or (programmed death-ligand 1) or (anti-PD-1) or (anti-PD-L1) or (CTLA-4) or (Cytotoxic T-lymphocyte antigen 4)).tw.
#3	#1 OR #2
#4	((Carcinoma) or (Neoplasms) or (Cancer) or (Tumour) or (Tumor)).tw.
#5	#3 and #4
#6	((mutation burden) or (mutational burden) or (mutation load) or (mutational load) or (TMB) or (TML)).tw.
#7	#5 and #6

For peer review only - http://bmjopen.bmj.com/site/about/guidelines.xhtml

Abbreviations:

ICIs= Immune Checkpoint Inhibitors

TMB = Tumor Mutation Burden

OR = Odds Ratio

ORR = Objective Response Rate /Overall Response Rate (ORR)

HR = Hazard Ratio

OS = Overall Survival

PFS = Progression-free Survival

DFS = Disease-free Survival

RFS = Recurrence-free Survival

DSS = Disease-specific Survival

SCLC = Small Cell Lung Cancer

FDA = U.S. Food and Drug Administration

CTLA-4 = Cytotoxic T Lymphocyte-associated Antigen-4

dMMR = Oncogenic Driver Mutations and Mismatch Repair Deficiency

PD-L1 = Programmed Cell Death Ligand 1

PD-1 = Programmed Cell Death 1

TILs = Tumorinfiltrating Lymphocytes

CI = Confidence Interval

NOS = Newcastle-Ottawa Scale

WES = Whole Exome Sequencing

MHC = Major Histocompatibility Complexes

RCT = Randomized Controlled Trial.

For peer review only

Search strategies and results of 5 databases

Database: PubMed, OVID, Embase, the Cochrane Central Register of Controlled Trials Library databases, Web of Science.

Data Run: 01/07/2022

database	Search strategies	results
Pubmed	#1 "Immune Checkpoint Inhibitors" [Mesh Terms]	6109
	#2 "immunotherap*"[Title/Abstract] OR "immune checkpoint inhibit*"[Title/Abstract] OR "ICI"[Title/Abstract] OR "ICIs"[Title/Abstract] OR "immune checkpoint block*"[Title/Abstract] OR "ICB"[Title/Abstract] OR "ICBs"[Title/Abstract] OR "pembrolizumab"[Title/Abstract] OR "avelumab"[Title/Abstract] OR "nivolumab"[Title/Abstract] OR "durvalumab"[Title/Abstract] OR "tremelimumab"[Title/Abstract] OR "atezolizumab"[Title/Abstract] OR "Ipilimumab"[Title/Abstract] OR "Cemiplimab"[Title/Abstract] OR "tiragolumab"[Title/Abstract] OR "Dostarlimab*"[Title/Abstract] OR "Camrelizumab"[Title/Abstract] OR "PD-1"[Title/Abstract] OR "programmed death 1"[Title/Abstract] OR "PD-L1"[Title/Abstract] OR "programmed death-ligand 1"[Title/Abstract] OR "PD-1/PD-L1"[Title/Abstract] OR "anti–PD-1/anti–PD-L1"[Title/Abstract] OR "CTLA-4"[Title/Abstract] OR "Cytotoxic T-lymphocyte antigen 4"[Title/Abstract]	164405
	#3 #1 OR #2	164676
	#4 "Carcinoma"[Title/Abstract] OR "Neoplasms"[Title/Abstract] OR "Cancer"[Title/Abstract] OR "Tumour" [Title/Abstract] OR "Tumor"[Title/Abstract]	3285875
	#5 #3 and #4	102540
	#6 "mutation burden"[Title/Abstract] OR "mutational burden"[Title/Abstract] OR "mutation load"[Title/Abstract] OR "mutational load"[Title/Abstract] OR "TMB"[Title/Abstract] OR "TML"[Title/Abstract]	10061
	#7 #5 and #6	3268
OVID	#1 exp Immune Checkpoint Inhibitors/	14572
	#2 ((immunotherap*) or (immune checkpoint inhibit*) or (ICI) or (immune checkpoint inhibit*) or (ICIs) or (immune checkpoint block*) or (ICB) or (ICBs) or (pembrolizumab) or (avelumab) or (nivolumab) or (durvalumab) or (tremelimumab) or (atezolizumab) or (Ipilimumab) or (Cemiplimab) or (tiragolumab) or (Dostarlimab) or (Camrelizumab) or (PD-1) or (programmed death 1) or (PD-L1) or (programmed death-ligand 1) or (anti-PD-1) or (anti-PD-L1) or (CTLA-4) or (Cytotoxic T-lymphocyte antigen 4)).tw.	153834
	#3 #1 OR #2	156787
	#4 ((Carcinoma) or (Neoplasms) or (Cancer) or (Tumour) or (Tumor)).tw.	3136257
	#5 #3 and #4	94825
	#6 ((mutation burden) or (mutational burden) or (mutation load) or (mutational load) or (TMB) or (TML)).tw.	9467
	#7 #5 and #6	2963
Embase	#1 'immunotherap*:ab,ti OR 'immune checkpoint inhibit*':ab,ti OR 'ici':ab,ti OR 'icis':ab,ti OR 'immune checkpoint block*':ab,ti OR 'icb':ab,ti OR 'icbs':ab,ti OR 'pembrolizumab':ab,ti OR 'avelumab':ab,ti OR 'nivolumab':ab,ti OR 'durvalumab':ab,ti OR 'tremelimumab':ab,ti OR 'atezolizumab':ab,ti OR 'ipilimumab':ab,ti OR 'cemiplimab':ab,ti OR 'tiragolumab':ab,ti OR 'dostarlimab*':ab,ti OR 'camrelizumab':ab,ti OR 'pd-1':ab,ti OR 'programmed death 1':ab,ti OR 'pd-l1':ab,ti OR 'programmed death-ligand 1':ab,ti OR 'pd-1/pd-l1':ab,ti OR 'anti–pd-1/anti–pd-l1':ab,ti OR 'ctla-4':ab,ti OR 'cytotoxic t-lymphocyte atigen 4':ab,ti	251032
	#2 'Carcinoma':ab,ti OR 'Neoplasms':ab,ti OR 'Cancer':ab,ti OR 'Tumour' ab,ti OR 'Tumor':ab,ti	1809315
	#3 #1 and #2	101253
	#4 'mutation burden':ab,ti OR 'mutational burden':ab,ti OR 'mutation load':ab,ti OR 'mutational load':ab,ti OR 'TMB':ab,ti OR 'TML':ab,ti	16290
	#5 #3 and #4	5654
Cochrane	#1 ("immunotherap* OR immune checkpoint inhibit* OR ICI OR ICIs OR immune checkpoint block* OR ICB OR ICBs OR pembrolizumab OR avelumab OR nivolumab OR durvalumab OR tremelimumab OR atezolizumab OR Ipilimumab OR Cemiplimab OR tiragolumab OR Dostarlimab* OR Camrelizumab	21080

For peer review only

	OR PD-1 OR programmed death 1 OR PD-L1 OR programmed death-ligand 1 OR PD-1/PD-L1 OR anti–PD-1/anti–PD-L1 ORCTLA-4 OR Cytotoxic T-lymphocyte antigen 4):ti,ab,kw<br>#2 (Carcinoma OR Neoplasms OR Cancer OR Tumour OR Tumor):ti,ab,kw<br>#3 #1 and #2<br>#4 (mutation burden OR mutational burden OR mutation load OR mutational load OR TMB OR TML):ti,ab,kw<br>#5 #3 and #4	 242648<br>13396<br>1495<br>460
web of science	#1 (TI=("immunotherap*" OR "immune checkpoint inhibit*" OR "ICI" OR "ICIs" OR "immune checkpoint block*" OR "ICB" OR "ICBs" OR "pembrolizumab" OR "avelumab" OR "nivolumab" OR "durvalumab" OR "tremelimumab" OR "atezolizumab" OR "Ipilimumab" OR "Cemiplimab" OR "tiragolumab" OR "Dostarlimab*" OR "Camrelizumab" OR "PD-1" OR "programmed death 1" OR "PD-L1" OR "programmed death-ligand 1" OR "PD-1/PD-L1" OR "anti–PD-1/anti–PD-L1" OR "CTLA-4" OR "Cytotoxic T-lymphocyte atigen 4")) OR AB=("immunotherap*" OR "immune checkpoint inhibit*" OR "ICI" OR "ICIs" OR "immune checkpoint block*" OR "ICB" OR "ICBs" OR "pembrolizumab" OR "avelumab" OR "nivolumab" OR "durvalumab" OR "tremelimumab" OR "atezolizumab" OR "Ipilimumab" OR "Cemiplimab" OR "tiragolumab" OR "Dostarlimab*" OR "Camrelizumab" OR "PD-1" OR "programmed death 1" OR "PD-L1" OR "programmed death-ligand 1" OR "PD-1/PD-L1" OR "anti–PD-1/anti–PD-L1" OR "CTLA-4" OR "Cytotoxic T-lymphocyte antigen 4")<br>#2 (TS = ("Carcinoma" OR " Neoplasms" OR " Cancer" OR " Tumour" OR " Tumor")) OR AB = ("Carcinoma" OR " Neoplasms" OR " Cancer" OR " Tumour" OR " Tumor")<br>#3 #1 and #2<br>#4 (TS = ("mutation burden" OR "mutational burden" OR "mutation load" OR "mutational load" OR "TMB" OR "TML")) OR AB = ("mutation burden" OR "mutational burden" OR "mutation load" OR "mutational load" OR "TMB" OR "TML")<br>#5 #3 and #4	191683<br>4045332<br>111505<br>13058<br>3510

For peer review only

PRISMA-P (Preferred Reporting Items for Systematic review and Meta-Analysis Protocols) 2015 checklist: recommended items to address in a systematic review protocol*

Section and topic	Item No	Checklist item	Location where item is reported
**ADMINISTRATIVE INFORMATION**			
Title:			
Identification	1a	Identify the report as a protocol of a systematic review	1
Update	1b	If the protocol is for an update of a previous systematic review, identify as such	1
Registration	2	If registered, provide the name of the registry (such as PROSPERO) and registration number	2
Authors:			
Contact	3a	Provide name, institutional affiliation, e-mail address of all protocol authors; provide physical mailing address of corresponding author	1
Contributions	3b	Describe contributions of protocol authors and identify the guarantor of the review	7
Amendments	4	If the protocol represents an amendment of a previously completed or published protocol, identify as such and list changes; otherwise, state plan for documenting important protocol amendments	3
Support:			
Sources	5a	Indicate sources of financial or other support for the review	7
Sponsor	5b	Provide name for the review funder and/or sponsor	7
Role of sponsor or funder	5c	Describe roles of funder(s), sponsor(s), and/or institution(s), if any, in developing the protocol	None
**INTRODUCTION**			
Rationale	6	Describe the rationale for the review in the context of what is already known	3
Objectives	7	Provide an explicit statement of the question(s) the review will address with reference to participants, interventions, comparators, and outcomes (PICO)	3,4
**METHODS**			
Eligibility criteria	8	Specify the study characteristics (such as PICO, study design, setting, time frame) and report characteristics (such as years considered, language, publication status) to be used as criteria for eligibility for the review	4
Information sources	9	Describe all intended information sources (such as electronic databases, contact with study authors, trial registers or other grey literature sources) with planned dates of coverage	4,5
Search strategy	10	Present draft of search strategy to be used for at least one electronic database, including planned limits, such that it could be repeated	10

For peer review only

Study records:			
Data management	11a	Describe the mechanism(s) that will be used to manage records and data throughout the review	5
Selection process	11b	State the process that will be used for selecting studies (such as two independent reviewers) through each phase of the review (that is, screening, eligibility and inclusion in meta-analysis)	5
Data collection process	11c	Describe planned method of extracting data from reports (such as piloting forms, done independently, in duplicate), any processes for obtaining and confirming data from investigators	5
Data items	12	List and define all variables for which data will be sought (such as PICO items, funding sources), any pre-planned data assumptions and simplifications	5
Outcomes and prioritization	13	List and define all outcomes for which data will be sought, including prioritization of main and additional outcomes, with rationale	4
Risk of bias in individual studies	14	Describe anticipated methods for assessing risk of bias of individual studies, including whether this will be done at the outcome or study level, or both; state how this information will be used in data synthesis	5
Data synthesis	15a	Describe criteria under which study data will be quantitatively synthesised	5
	15b	If data are appropriate for quantitative synthesis, describe planned summary measures, methods of handling data and methods of combining data from studies, including any planned exploration of consistency (such as I^2, Kendall's τ)	5,6
	15c	Describe any proposed additional analyses (such as sensitivity or subgroup analyses, meta-regression)	5,6
	15d	If quantitative synthesis is not appropriate, describe the type of summary planned	None
Meta-bias(es)	16	Specify any planned assessment of meta-bias(es) (such as publication bias across studies, selective reporting within studies)	5
Confidence in cumulative evidence	17	Describe how the strength of the body of evidence will be assessed (such as GRADE)	None

*** It is strongly recommended that this checklist be read in conjunction with the PRISMA-P Explanation and Elaboration (cite when available) for important clarification on the items. Amendments to a review protocol should be tracked and dated. The copyright for PRISMA-P (including checklist) is held by the PRISMA-P Group and is distributed under a Creative Commons Attribution Licence 4.0.**

From: Shamseer L, Moher D, Clarke M, Ghersi D, Liberati A, Petticrew M, Shekelle P, Stewart L, PRISMA-P Group. Preferred reporting items for systematic review and meta-analysis protocols (PRISMA-P) 2015: elaboration and explanation. BMJ. 2015 Jan 2;349(jan02 1):g7647.

For peer review only
